# Subcellular proteomic characterization of the high-temperature stress response of the cyanobacterium *Spirulina platensis*

**DOI:** 10.1186/1477-5956-7-33

**Published:** 2009-09-02

**Authors:** Apiradee Hongsthong, Matura Sirijuntarut, Rayakorn Yutthanasirikul, Jittisak Senachak, Pavinee Kurdrid, Supapon Cheevadhanarak, Morakot Tanticharoen

**Affiliations:** 1 BEC Unit, National Center for Genetic Engineering and Biotechnology, 83 Moo8, Thakham, Bangkhuntien, Bangkok 10150, Thailand; 2 School of Bioresources and Technology; King Mongkut's University of Technology Thonburi, 83 Moo8, Thakham, Bangkhuntien, Bangkok 10150, Thailand; 3 Pilot Plant Development and Training Institute; King Mongkut's University of Technology Thonburi, 83 Moo8, Thakham, Bangkhuntien, Bangkok 10150, Thailand

## Abstract

The present study examined the changes in protein expression in *Spirulina platensis *upon exposure to high temperature, with the changes in expression analyzed at the subcellular level. In addition, the transcriptional expression level of some differentially expressed proteins, the expression pattern clustering, and the protein-protein interaction network were analyzed. The results obtained from differential expression analysis revealed up-regulation of proteins involved in two-component response systems, DNA damage and repair systems, molecular chaperones, known stress-related proteins, and proteins involved in other biological processes, such as capsule formation and unsaturated fatty acid biosynthesis. The clustering of all differentially expressed proteins in the three cellular compartments showed: (*i*) the majority of the proteins in all fractions were sustained tolerance proteins, suggesting the roles of these proteins in the tolerance to high temperature stress, (*ii*) the level of resistance proteins in the photosynthetic membrane was 2-fold higher than the level in two other fractions, correlating with the rapid inactivation of the photosynthetic system in response to high temperature. Subcellular communication among the three cellular compartments via protein-protein interactions was clearly shown by the PPI network analysis. Furthermore, this analysis also showed a connection between temperature stress and nitrogen and ammonia assimilation.

## Introduction

High temperature stresses are well known to cause protein aggregation and denaturation, and in order to cope with these stress, a cellular response occurs. Proteomics research regarding cellular responses to high temperature stresses was carried out in bacteria. The majority of the differentially expressed proteins belong to the group of proteins including heat shock responsive chaperones and proteases, of which many are also induced in response to gamma irradiation and/or desiccation [1,2]. In addition to these proteins, some central metabolic proteins were also found by Fourier transform ion cyclotron resonance (FTIR) mass spectrometric proteomics analysis [2]. Thus, it was hypothesized that elevated temperature may induce a general stress response, and could lead to cross-protection against related stresses [2].

In cyanobacteria, gene regulation mediated by high temperature stresses has been studied less extensively than regulatory responses to low temperature stresses. In some cyanobacteria, such as *Synechocystis*, *Synechococcus *and *Nostoc*, heat shock responses have been investigated [3-5]. An alternative sigma factor (SigH) and heat-shock protein (HSP) were both significantly induced immediately following exposure to heat stress [6,7].

Since *Spirulina *cells are grown in outdoor ponds for mass cultivation, they are exposed to various stress conditions, including high temperature stress. During daylight hours in tropical countries, the cells are exposed to high temperatures of around 40°C. The temperature fluctuation in outdoor mass cultivation has a serious effect on biomass yield and the biochemical content of the cells. Some components of *Spirulina *cells have pharmaceutical benefits, such as unsaturated fatty acids. The level of unsaturated fatty acids in membrane lipids has been shown to play a critical role in response to temperature change in various organisms. Substantial evidence points to an association between fatty acid desaturation and temperature stress [8]. Due to this relationship, the molecular responses to high temperature stress of genes involved in the desaturation process have been well studied in *Spirulina*. Upon temperature increase from 35°C to 40°C, the level of the polyunsaturated fatty acid γ-linolenic acid (GLA) in *Spirulina plantensis *decreases approximately 30%, compared to the level found in cells grown at an optimal temperature (35°C) [9]. This highlights the regulation of *Spirulina*-Δ^6 ^desaturase, which carries out the last step of the *Spirulina *desaturation process. Thus, the transcriptional levels of the three *Spirulina*-desaturase genes, *desC*, *desA *and *desD*, were examined [9].

Despite the heat shock response studies in other cyanobacteria, transcriptomic and proteomic analyses of responses to high temperature stress have not been performed in *Spirulina*. The lack of a complete *Spirulina *genome sequence hinders this relevant research. Therefore, the present study focused on the *S. platensis *response to a temperature upshift at the subcellular level. This analysis was performed by proteomic and transcriptomic analyses, protein clustering (based on protein expression patterns), and protein-protein interaction analysis.

## Materials and methods

### Organisms and culture conditions

*S. platensis *strain C1 cultures were grown at 35°C under illumination by a 100 μEm^-2^s^-1 ^fluorescent light with continuous stirring in 2 L of Zarrouk's medium [10]. The culture was grown until the optical density at 560 nm reached 0.4 (mid-log phase), and subsequently a cell sample was harvested by filtration before shifting the growth temperature (t = 0 min). The growth temperature was then immediately shifted from 35°C to 40°C and the culture was incubated for 45, 90, or 180 min before cell harvesting.

### Sample preparation

The harvested cells were washed and lysed as described previously [11]. The three subcellular fractions of *Spirulina *were separated according to the methods described by Murata and Omata, and Hongsthong et al. [11,12]. It should be noted that the soluble fraction contained cytoplasmic and periplasmic proteins. The purity of thylakoid (TM) and plasma membrane (PM) fractions were tested by scanning absorption spectra and western blot analysis as described previously [11]. The membrane pellet was resuspended in 500 μl of dissolving buffer, containing 2 M thiourea, 8 M urea, 20 mM Tris, 30 mM DTT, 1% (v/v) IPG buffer, 0.05% (w/v) β-dodecyl maltoside, and 4% (w/v) CHAPS, prior to protein precipitation using a 2D-clean up kit (GE Healthcare Biosciences, USA). The protein pellets were then dissolved in dissolving buffer without DTT before determining protein concentrations using a 2D-Quant kit protein assay (GE Healthcare Biosciences).

### Protein separation by two-dimensional differential gel electrophoresis (2D-DIGE) and protein profile analyses

The pH of the protein samples was adjusted to 8.5 and 10 μg of each sample, prepared as described above, was labeled with fluorescent dyes, according to the manufacturer's instructions (GE Healthcare Biosciences). The proteins were separated by 2D-DIGE and statistically analyzed for differential expression as described previously [13]. Protein spot picking and in-gel digestion of the proteins of interest were carried out as described [13].

To study phosphorylated proteins, 2D-PAGE using 7 cm non-linear IPG strips, pH 3-10 and 4-7 (GE Healthcare Biosciences), in the first dimension were performed. Subsequently, the second dimension was conducted as described above, followed by western blot analysis. Three independent experiments were performed.

### Protein identification using MALDI-TOF mass spectrometry

After protein digestion with trypsin, peptide samples were analyzed by MALDI-TOF mass spectrometry. For protein identification, the resulting peptide mass fingerprints (PMFs) were analyzed with our in-house software tool, using the unpublished *S. platensis *C1 database, which was generated from *in silico *digestion of the *S. platensis *C1 completed genome sequence. The search parameters and data filtering were carried out as described in detail previously [13]. PMF identification results of a protein spot were required to be reproducible in order to consider the spot as an identified protein.

### Western blot analysis

Following 2D-PAGE, detection of phosphorylated proteins was performed by western blot analysis [14]. Phosphorylations on serine, threonine, and tyrosine residues were detected separately using monoclonal antibodies raised against the designated phosphorylated amino acid residues (Santa Cruz, USA, and Assay Designs, USA). Phosvidin and trypsin inhibitor were used as positive and negative controls, respectively. An equal amount of each protein sample was separated by 2D-PAGE, as described previously, and transferred onto a nitrocellulose membrane using a semi-dry electroblotter at a constant voltage of 20 V for 30 min at room temperature. A chemiluminescent western blot detection kit, with an HRP-based system and chemiluminescent molecular weight markers, was used (Pierce, USA) according to the manufacturer's instructions.

The resulting phosphoproteome spot-maps were then matched with the 2D-DIGE spot-maps over the same pH range. Finally, the analysis of differentially expressed proteins containing phosphorylated amino acid residues was performed.

### Transcriptional analysis by RT-PCR

The details of *Spirulina *RNA isolation were described previously [13]. The transcriptional expression levels of the genes of interest were analyzed by RT-PCR using an AccessQuick™ RT-PCR System (Promega, USA). The RT-PCR analysis was carried out according to the manufacturer's instructions. Details on primers are shown [see Additional file 1]. The densities of the RT-PCR product bands were quantified using the Image Quant TL program (GE Healthcare Biosciences). Normalization of the RT-PCR product levels was performed by comparing the density of the designated band to the density of the 16S rRNA band.

### Protein clustering based on expression patterns

The protein expression dataset was validated for the input well-form of protein ratio values. The null values and those ratios that were extremely high or low, relative to the threshold value of 1e+-10, were filtered out. K-mean clustering was applied to obtain 23 profiles of the protein expression patterns. A good k-profile number was chosen by simulation, as described by Martin et al. [15].

### Potential protein-protein interaction network

A protein-protein interaction (PPI) network in *Spirulina *was constructed on the prototype PPI database of *Synechocystis *from CyanoBase [16]. Prototype construction is based on a graph in which nodes and edges represent proteins and interactions, respectively. Each interaction was experimentally identified by the yeast two-hybrid system. Source nodes represent the bait proteins, and prey proteins are represented by the target nodes. Next, homologous proteins, identified by BLAST similarity searches with significant values less than 1e-10, were mapped to their best-hit *Synechocystis *protein nodes. Finally, differentially expressed proteins in *Spirulina *were mapped to their corresponding nodes. The size of each node represents the level of differential expression.

## Results and Discussion

In the present study, 2D-DIGE [see fig S1, S2 and S3; Additional file 2] and mass spectrometry were employed to identify differentially expressed proteins at the subcellular level of *Spirulina*, in response to a temperature increase from 35°C to 40°C.

### Differentially expressed proteins

#### Up- regulated proteins

Expression profile analysis identified 38, 50 and 26 up-regulated proteins in the PM, soluble, and TM fractions, respectively. Of these, 2, 6 and 2 proteins were phosphorylated in the three respective fractions (Tables 1, 2 and 3). Further analysis showed that several of the up-regulated proteins are involved in two-component signal transduction: histidine kinase, Ser/Thr protein kinase and response regulator, including GGDEF (Gly-Gly-Asp-Glu-Phe) domains. It should be noted that a large number of these proteins were detected in the soluble fraction, suggesting the dissociation of these domains from the membrane-bound domains of the two-component systems during sample preparation. Moreover, our study showed phosphorylation of the GGDEF domain in the soluble fraction, consistent with the report by Ryjenkov et al. [17]. Phosphorylation of this domain is required for its activity. Thus, the GGDEF domains represent the output of complex bacterial signal transduction networks, which convert different signals into the production of a secondary messenger, cyclic diguanylic acid [17,18]. In addition, it has been reported that the GGDEF domain plays a critical role in heterocyst formation [19].

**Table 1:** Significantly up-regulated proteins identified in the plasma membrane fraction after the immediate temperature upshift.

				**theoretical**		**experimental**				
**spot#**	**orf**	**protein name**	**%cov**	**pI**	**MW (kDa)**	**pI**	**MW (kDa)**	fold*	**t-test**	**cluster**^t^
**Up regulated proteins **(separated by pH range 3-10 in the first dimension)										
*Two component systems*										
357	AP07650006	Two-component hybrid sensor and regulator	6.47	4.97	146.95	5.71	265.61	1.43	0.044	5
518	AP07580004	Two-component sensor histidine kinase	8.57	5.26	133.55	5.36	239.78	1.78	0.0059	23
793	AP06360005	Two component hybrid sensor and regulator	6.9	5.41	159.88	6.4	188.54	2.47	0.013	14
808	AP07880008	Two-component hybrid sensor and regulator	2.86	4.97	200.24	6.17	183.78	3.15	0.025	15
872	AP07580004	Two-component sensor histidine kinase	5.94	5.26	133.55	5.28	174.61	1.89	0.027	23
1097	AP06990006	Hybrid sensor and regulator	7.34	5.5	104.01	5.88	140.14	1.43	0.047	23
1102	AP07670017	Two-component sensor histidine kinase	6.57	4.88	124.16	4.58	139.07	1.96	0.034	23
1147	AP07670017	Two-component sensor histidine kinase	8.51	4.88	124.16	4.65	130.45	1.77	0.041	23
1241	AP07670017	Two-component sensor histidine kinase	5.92	4.88	124.16	4.48	120.51	3.49	0.019	15
1564	AP07970028	Two-component system sensory histidine kinase	6.22	4.8	108.09	4.49	90.03	1.71	0.016	23
1592	AP04840005	Serine/threonine kinase with TPR repeat	7.45	8.37	82.39	5.24	86.42	1.81	0.043	23
2406	AP07830002	Sensory box/GGDEF family protein	8.15	5.38	53.01	4.51	39.31	1.78	0.016	9
*Stress related proteins*										
508	AP06620005	Glycosyl transferase, family 2:Glycosyl transferase, group 1	4.14	6.57	128.59	5.29	239.78	1.82	0.025	23
526	AP06740013	Ferredoxin-glutamate synthase	5.87	5.6	169.95	5.43	238.56	2.2	0.042	14
532	AP05380002	DEAD/DEAH box helicase domain protein (membrane-helicase)	5.29	6.09	239.75	5.5	236.73	2.24	0.0014	14
821	AP05300003	ATPase of the ABC class	9.74	5.46	66.65	6.63	184.25	2.18	0.0093	23
874^p^	AP05380002	DEAD/DEAH box helicase domain protein (membrane-helicase)	4.81	6.09	239.75	5.48	175.06	1.95	0.013	23
995	AP07470001	RNA polymerase sigma-70 factor	19.07	9.81	30.04	5.56	154.44	1.76	0.024	23
1028	AP06740013	Ferredoxin-glutamate synthase	6.89	5.6	169.95	4.47	151.31	2.09	0.011	23
1029	AP08060123	Putative transcriptional regulator, LysR family	4.49	6.61	34.76	4.57	148.63	2.11	0.035	23
1277	AP05380002	DEAD/DEAH box helicase domain protein (membrane-helicase)	3.9	6.09	239.75	5.48	116.27	1.99	0.026	23
1284	AP06510003	Putative membrane carboxypeptidase	5.77	8.68	84.38	5.68	115.68	1.65	0.039	23
1388	AP06740013	Ferredoxin-glutamate synthase	4.47	5.6	169.95	7.08	105.50	2	0.023	18
2187	AP04600003	S-adenosyl-L-homocysteine hydrolase	10.27	5.63	48.77	4.52	48.24	1.57	0.037	9
*DNA damage/DNA repairing system*										
1479	AP07790020	Type II site-specific deoxyribonuclease	5.03	6.85	35.22	6.53	97.71	3.88	0.021	15
1547	AP02010002	Restriction endonuclease	11.57	5.26	53.91	5.66	91.66	1.69	0.031	14
*Proteins containing conserved motifs*										
422	AP07330005	TPR repeat- peptidase M, neutral zinc metallopeptidase, zinc-binding site	4.54	8.84	95.92	3.65	253.01	1.38	0.02	9
571	AP06390003	WD-40 repeat protein- transcriptional regulator, XRE family	5.52	6.2	160.34	3.48	222.64	3.11	0.048	22
943	AP07740012	TPR repeat-glycosyl transferase/capsule polysaccharide biosynthesis	2.95	5.59	103.58	5.27	161.30	1.99	0.032	14
1274	AP05550005	TPR repeat-putative prenyltransferase/glycosyl transferase	12.7	5.34	41.12	4.58	118.07	1.45	0.025	9
1817	AP06930009	TPR repeat-O-linked GlcNAc transferase	3.65	5.94	71.42	6.33	70.07	1.81	0.018	23
1983	AP05550005	TPR repeat-putative prenyltransferase/glycosyl transferase	12.7	5.34	41.12	6.35	61.19	1.8	0.013	14
2733^p^	AP05550005	TPR repeat-putative prenyltransferase/glycosyl transferase	12.7	5.34	41.12	4.93	28.19	1.74	0.049	9
*Others*										
611	AP06080002	Putative enzyme of poly-gamma-glutamate biosynthesis, capsule formation	5.68	5.59	72.57	5.83	221.50	1.9	0.031	14

**Up regulated proteins **(separated by pH range 4-7 in the first dimension)										
*Two component system*										
2386	AP01720004	Putative two-component sensor histidine kinase	6.34	5.25	57.98	5.08	53.46	2.21	0.038	18
*Stress related proteins*										
1569	AP05860006	Putative glycosyl transferase	9.08	5.62	132.60	4.84	96.48	1.89	0.031	23
2179	AP07990044	Putative ABC transporter	6.92	8.57	88.65	5.74	62.93	2.4	0.011	14
*Translation machinery*										
1477	AP07620038	50S ribosomal protein L4	13.81	10.04	23.34	5.08	103.76	2.81	0.02	14

* Fold represents fold change value which is volume ratio of after the temperature upshift (180 min)/before the temperature upshift. Volume ratio refers to the ratio of the normalized volumes of a pair of spots (the same spot of before and after the temperature upshift), for example, a value of 2.0 represents a two-fold increase while -2.0 represents a two-fold decrease.

^p ^These protein spots are phosphorylated at Ser, Thr and Tyr residues, detected by western blot analysis before the temperature upshift (0 min) and after the temperature upshift (45, 90, 180 min).

^t ^Cluster types; (i) cluster 5, 14,15,18 and 23 are sustained tolerance proteins (ii) cluster 22 is adaptation proteins and (iii) cluster 9 and 14 are undetermined-pattern proteins.

**Table 2:** Significantly up-regulated proteins identified in the soluble fraction after the immediate temperature upshift.

				**theoretical**		**experimental**				
**spot#**	**orf**	**protein name**	**%cov**	**pI**	**MW (kDa)**	**pI**	**MW (kDa)**	fold*	**t-test**	**cluster**^t^
**Up regulated proteins **(separated by pH range 3-10 in the first dimension)										
*Two component systems*										
1282	AP07350018	Hybrid sensor and regulator	4.51	5.16	157.04	9.24	125.42	5.21	0.047	22
1399	AP08000025	Two-component sensor histidine kinase	8.04	5.09	80.65	8.67	116.52	3.42	0.022	4
2377	AP07580004	Two-component sensor histidine kinase	7.38	5.26	133.55	7.69	51.45	1.51	0.012	16
2765	AP07580004	Two-component sensor histidine kinase	4.07	5.26	133.55	8.13	34.68	4.05	0.0035	4
*Stress related proteins*										
2693	AP04260002	NADPH-dependent FMN reductase	7.48	6.53	24.75	3.37	37.83	2.98	0.039	5
*Translation machinery*										
23	AP07220011	30S ribosomal protein S1	14.32	4.48	42.60	4.88	308.16	1.55	0.037	16
*Hypothetical proteins*										
410	AP07020011	Conserved hypothetical protein	2.74	5.46	249.83	3.61	228.97	2.86	0.026	1
*Proteins containing conserved motif*										
1736	AP06120011	TPR repeat-containing protein-O-linked GlcNAc transferase	5.79	4.88	72.71	3.76	91.01	1.54	0.05	16

**Up regulated proteins **(separated by pH range 4-7 in the first dimension)										
*Two component systems*										
599^p^	AP03710004	Sensory box/GGDEF family protein	11.55	4.95	65.15	5.16	226.88	4.41	0.034	4
665	AP06710002	Multi-sensor signal transduction histidine kinase	5.45	5.25	116.20	5.03	220.07	1.75	0.047	6
727	AP07310007	Hybrid sensory kinase	2.82	5.34	100.91	5.13	209.99	1.87	0.039	19
735	AP06460007	Multi-sensor Hybrid Histidine Kinase	6.58	4.93	200.70	5.21	210.48	1.73	0.039	1
737	AP02950002	Putative response regulator receiver signal transduction histidine kinase	13.19	4.91	42.67	5.27	209.99	2.04	0.0073	10
788	AP08040014	Ethylene response sensor protein	6.1	4.92	120.25	5.32	203.21	5.64	0.0035	2
1072	AP04840005	Serine/threonine kinase with TPR repeat	7.03	8.37	82.39	5.92	172.45	1.57	0.026	6
1153	AP07580004	Two-component sensor histidine kinase	5.85	5.26	133.55	5.75	159.61	1.54	0.025	6
1343	AP07350018	Hybrid sensor and regulator	2.25	5.16	157.04	5.73	142.96	1.34	0.02	6
1357	AP07580004	Two-component sensor histidine kinase	2.37	5.26	133.55	4.9	139.65	3.73	0.0091	4
1377	AP07580004	Two-component sensor histidine kinase	5.51	5.26	133.55	5.21	137.37	3.29	0.022	5
1482	AP07670017	Two-component sensor histidine kinase	4.07	4.88	124.16	5.59	127.74	1.7	0.021	6
1787	AP07430015	Sensory box histidine kinase/response regulator	1.21	5.41	138.91	4.56	102.00	2.01	0.0012	19
1883^p^	AP06710002	Multi-sensor signal transduction histidine kinase	5.74	5.25	116.20	5.94	94.40	1.48	0.021	6
2635	AP04840005	Serine/threonine kinase with TPR repeat	5.79	8.37	82.39	5.31	50.01	1.36	0.016	6
2940	AP08000025	Two-component sensor histidine kinase	4.23	5.09	80.65	5.2	38.73	1.42	0.035	6
*Stress related proteins*										
460	AP07250004	Glycosyl transferase, family 2	1.19	5.45	236.54	5.34	248.02	2.16	0.0088	8
512^p^	AP07620006	ABC transporter-like protein	8.96	5.5	64.24	5.3	238.33	3.11	0.01	4
613	AP07180022	Putative aldehyde dehydrogenase	12.21	6.38	29.30	5.36	226.35	4.15	0.021	4
799	AP06960005	Fe-S oxidoreductase	4.58	5.83	60.23	5.21	202.26	4.87	0.017	4
1328	AP07510011	Ribitol type dehydrogenase protein	5.1	5.53	47.30	5.96	143.97	2.11	0.013	1
1753	AP06510003	Putative membrane carboxypeptidase	3.15	8.68	84.38	5.46	105.40	2.65	0.029	1
1835	AP07620006	ABC transporter-like protein	8.96	5.5	64.24	4.76	99.17	2.22	0.00083	10
1901^p^	AP05290001	Putative transposase	12.64	9.92	40.43	6.11	92.87	1.43	0.0093	6
*Chaperones*										
1053	AP08040017	Chaperonin GroEL (HSP60 family)	5.73	4.89	58.72	5.61	173.26	1.5	0.0059	6
1451	AP04730007	Chaperone clpB 2	7.22	5.4	98.73	5.17	131.08	1.98	0.008	10
*DNA damage/DNA reparing system*										
608^p^	AP06420003	RNA-directed DNA polymerase	7.92	10.27	50.25	5.27	225.29	4.45	0.028	4
1278	AP05970008	DNA gyrase subunit A	5.69	5.16	61.11	4.97	148.42	2.47	0.0019	10
1209	AP02770002	Putative chromosome segregation ATPases	9.45	5.2	38.26	5.31	155.55	1.47	0.033	6
1789	AP06960003	Putative exonuclease SbcC	3.05	4.89	76.57	5.79	103.20	1.33	0.00039	6
1969	AP06870011	Type I restriction system endonuclease	4.07	6.4	119.09	5	88.20	1.7	0.038	19
3100	AP07900024	Restriction endonuclease	9.39	5.89	24.50	5.76	34.05	2.49	0.043	10
*Translation machinery*										
1368	AP04960025	30S ribosomal protein S2	6.25	4.77	32.18	4.95	138.99	5.46	0.016	4
*Channeling systems*										
1488	AP07910035	Outer membrane efflux protein	6.03	4.89	76.57	5.71	127.74	1.62	0.017	6
*Hypothetical protein*										
520	AP07020011	Conserved hypothetical protein	1.48	5.46	249.83	4.8	235.55	1.44	0.026	6
1726	AP07780003	Conserved hypothetical protein	7.03	4.82	68.18	5.01	107.15	2.26	0.015	10
*Proteins containing conserved motifs*										
537^p^	AP06120011	TPR repeat-containing protein-O-linked GlcNAc transferase	5.01	4.88	72.71	5.42	234.45	1.49	0.032	19
1205	AP06120011	TPR repeat-containing protein-O-linked GlcNAc transferase	5.95	4.88	72.71	4.94	154.46	2.29	0.023	10
1212	AP06930009	TPR repeat-containing protein-O-linked GlcNAc transferase	6.66	5.94	71.42	5.38	155.55	1.5	0.0052	22
1358	AP06390003	WD-40 repeat protein-transcriptional regulator, XRE family	2.9	6.2	160.34	5.01	139.97	4.79	0.018	4
*Others*										
840	AP06080002	Putative enzyme of poly-gamma-glutamate biosynthesis, capsule formation	8.29	5.59	72.57	5.3	199.90	3.31	0.019	4

^t ^Cluster types; (i) cluster 1, 2, 6, 16 and 19 are sustained tolerance proteins and (ii) cluster 4, 5, 8, 10 and 22 are undetermined-pattern proteins.

**Table 3:** Significantly up-regulated proteins identified in the thylakoid membrane fraction after the immediate temperature upshift.

				**theoretical**		**experimental**				
**spot#**	**orf**	**protein name**	**%cov**	**pI**	**MW (kDa)**	**pI**	**MW (kDa)**	fold*	**t-test**	**cluster**^t^
**Up regulated proteins **(separated by pH range 3-10 in the first dimension)										
*Two component systems*										
954	AP05090011	Multi-sensor signal transduction histidine kinase	5.69	5.23	135.61	6.19	161.10	2.1	0.048	13
1011	AP07350018	Hybrid sensor and regulator	3.42	5.16	157.04	4.92	150.14	1.37	0.047	8
1707	AP06710002	Multi-sensor signal transduction histidine kinase	7.52	5.25	116.20	5.02	81.51	1.78	0.0043	22
*Chaperones*										
912	AP07830020	Molecular chaperone DnaK	7.38	4.78	68.30	3.52	168.46	1.91	0.0078	23
*Stress related proteins*										
571	AP07830017	Polyphosphate kinase	4.16	5.47	82.79	4.4	219.16	1.6	0.042	23
1078	AP07620006	ABC transporter-like protein	5.62	5.5	64.24	3.98	142.91	1.43	0.0079	8
1366	AP05380002	DEAD/DEAH box helicase domain protein (membrane-helicase)	2.67	6.09	239.75	4.14	111.67	1.88	0.011	23
3925	AP05940003	Transcriptional regulator, LysR family	9.86	7.11	31.25	8.37	6.19	2.66	0.012	23
*DNA repairing system*										
684	AP04930005	N-6 DNA methylase	8.7	5.24	59.46	3.32	206.66	2.23	0.039	23
1399^p^	AP05780004	Type I site-specific restriction-modification with related helicase system	10.55	5.97	127.07	7.33	107.30	1.59	0.022	8
*Translation machinery*										
1634	AP07840003	30S ribosomal protein S18	28.17	10.58	8.36	4.95	87.88	1.62	0.037	8
*Channeling system*										
1499	AP05960003	Preprotein translocase SecA subunit	6.67	5.14	105.74	5.8	98.14	1.88	0.023	22
*Hypothetical proteins*										
564	AP07810017	Hypothetical protein	12.75	7.9	211.37	4.3	221.75	1.6	0.048	22
*Proteins containing conserved motifs*										
1242	AP06700002	WD-40 repeat protein-peptidase C14, caspase catalytic subunit p20	4.37	5.26	183.10	4.13	122.68	1.68	0.019	23
1258	AP06390003	Pentapeptide repeat- transcriptional regulator, XRE family	10.82	6.2	160.34	5.43	121.53	1.64	0.0057	8
*Others*										
1627	AP04100001	Glucose-inhibited division protein A	5.49	6.21	71.63	4.83	88.29	1.79	0.039	8

**Up regulated proteins **(separated by pH range 4-7 in the first dimension)										
*Two component systems*										
1513^p^	AP06990006	Phytochrome-like protein	7.12	5.5	104.01	5.78	101.22	1.72	0.0076	23
1954	AP07670017	Two-component sensor histidine kinase	4.35	4.88	124.16	5.07	63.09	1.85	0.051	ud
*Stress related proteins*										
502	AP07700024	Putative transcriptional acitvator, Baf	15.77	6.59	28.74	5.73	232.21	2.37	0.04	21
701	AP06620002	Glycosyl transferase domain containing protein	7.29	5.92	134.25	5.03	205.94	1.69	0.025	23
781	AP08030034	Aldo/keto reductase	16.02	6.22	38.85	5.08	194.92	1.88	0.046	23
879	AP06740013	Ferredoxin-glutamate synthase	2.04	5.6	169.95	4.66	184.48	2.27	0.047	23
2082	AP04600003	S-adenosyl-L-homocysteine hydrolase	14.96	5.63	48.77	5.27	54.85	2.18	0.023	23
*DNA damage/DNA reparing system*										
163	AP03680003	Putative RNA-directed DNA polymerase (Reverse transcriptase):HNH endonuclease	8.13	9.73	56.22	5.1	293.02	3.89	0.027	6
*Proteins containing conserved motif*										
1852	AP05550005	TPR repeat-prenyl transferase	12.7	5.34	41.12	4.75	72.40	1.89	0.0071	23
*Others*										
452	AP07910008	Adenylate cyclase	13.72	5.59	51.10	5.42	237.50	5.53	0.032	21

ud means the protein expression pattern(s) cannot be clustered.

^t ^Cluster types; cluster 6, 8,13, 21, 22 and 23 are sustained tolerance proteins.

Three molecular chaperones were found to be up-regulated in two subcellular fractions, GroEL (Hsp60) and ClpB in the soluble fraction and DnaK (Hsp70) in the thylakoid membrane fraction. The major molecular chaperones, such as DnaK/DnaJ, GroES/GroEL and ClpB, are involved in *de novo *protein folding of newly synthesized polypeptides and solubilising aggregated proteins under high temperature stress conditions [20].

Although the chaperone proteins have been generally reported to be soluble proteins, membrane-bound chaperones have been identified in many eukaryotes and prokaryotes, including cyanobacteria. This type of chaperone has been proposed to play a role in protein translocation, translational machinery associated with the surface of the thylakoid membrane, and enhancement of membrane fluidity through association with membrane lipids in response to heat stress [21].

In the case of stress related proteins, glycosyl transferase and ABC transporter were detected in all subcellular fractions, while membrane helicase, LysR and ferredoxin-glutamate synthase were found in the membrane fractions (TM and PM). Glycosyl transferase has been reported to be involved in osmo- and thermoadaptation [22].

One up-regulated membrane protein, DEAD/DEAH box helicase, is involved in RNA maturation, proof-reading and enhancement of DNA-unwinding [23]. Interestingly, the helicase present in the PM of *Spirulina *was phosphorylated. Phosphorylation of RNA helicase is rare, and mostly found in plants. This modification is believed to be a direct link between helicase and environmental sensing-signal transduction phosphorylation cascades [23]. To the best of our knowledge, this is the first evidence of helicase phosphorylation in cyanobacteria.

A few of the proteins involved in DNA damage, repair and modification (endonucleases and methylases) were dramatically induced in the membrane fractions upon temperature upshift. Under stress conditions where DNA damage may occur, induction of SbcC is expected. This exonuclease removes unusual DNA structures, such as hairpins, that are generated upon DNA damage [24].

In contrast to cold stress conditions in *Spirulina*, the significant induction of DNA gyrase upon induction of heat stress could lead to elevated function of the DNA repair system [25]. Another up-regulated protein that plays a vital role in DNA replication, repair and chromosome stability is chromosome segregation ATPase [26,27]. These results demonstrate the requirement for DNA replication, modification and repair for cell survival under heat stress conditions in this cyanobacterium.

#### Down-regulated proteins

Two down-regulated proteins were identified in the PM and soluble fractions, while thirteen down-regulated proteins were identified in the TM fraction (Table 4).

**Table 4:** Significantly down-regulated proteins identified in the three subcellular fractions after the immediate temperature upshift.

					**theoretical**		**experimental**				
**spot#**	**frac/pH range**	**orf**	**protein name**	**%cov**	**pI**	**MW (kDa)**	**pI**	**MW (kDa)**	fold*	**t-test**	**cluster**^t^
**Plasma membrane fraction**											
2410	PM/4-7	AP07850026	Two-component hybrid sensor and regulator	6.97	5.13	143.09	4.81	52.64	-2	0.024	4
2459	PM/4-7	AP04660005	TPR repeat-hypothetical protein	7.89	6.2	33.86	4.74	51.95	-2.18	0.024	4
**Soluble fraction**											
1396	SOL/4-7	AP07820015	Putative WD-40 repeat protein	3.6	9.2	120.78	4.34	134.50	-1.55	0.01	18
2466	SOL/4-7	AP07540009	Two-component hybrid sensor and regulator	3.37	5.33	156.25	4.48	58.11	-1.8	0.019	8
**Thylakoid membrane fraction**											
*Two component systems*											
1193	TM/4-7	AP06440002	Twin-arginine translocation pathway signal	7.82	7.84	44.13	5.1	136.65	-2.12	0.051	ud
1781	TM/4-7	AP07540011	Two component response regulator	7.06	6.09	74.15	5.78	77.65	-1.54	0.028	14
2019	TM/4-7	AP06580007	Two-component hybrid sensor and regulator	6.94	4.36	127.39	5.64	58.10	-3.22	0.0092	17
*Stress related proteins*											
1393	TM/4-7	AP07870030	Molybdopterin oxidoreductase	11.14	8.29	82.17	5.07	112.15	-2.76	0.027	10
1539	TM/4-7	AP05060006	5-methyltetrahydropteroyltriglutamate-homocysteine S-methyltransferase	9.86	5.18	87.57	6.84	100.21	-1.79	0.00015	10
*DNA damage/DNA repairing system*											
1512	TM/3-10	AP07840004	Ribonuclease II	10.27	4.93	76.91	4.54	97.91	-1.3	0.049	14
1316	TM/4-7	AP07740021	Chromosome segregation ATPases	6.79	4.97	83.23	5.26	123.95	-1.41	0.047	1
1457	TM/4-7	AP07790019	Putative site-specific DNA-methyltransferase (cytosine-specific)	11.11	8.39	55.18	5.07	106.41	-4.43	0.0054	12
2943	TM/3-10	AP07750017	UvrD/REP helicase	4.39	5.47	121.28	7.49	18.08	-2.01	0.003	10
*Proteins containing conserved motif*											
2219	TM/4-7	AP07230009	TPR repeat containing protein-hypothetical protein	16.61	4.72	31.94	6.44	47.21	-2.7	0.035	10
*Others*											
1301	TM/4-7	AP06400004	Slr0554 protein.	7.41	8.54	108.70	7.11	124.89	-6.45	0.02	17
2577	TM/3-10	AP06920007	Aldehyde-alcohol dehydrogenase	13.21	5.85	54.75	7.58	30.60	-1.83	0.046	10
3572	TM/4-7	AP07900036	Delta-9 desaturase	7.78	7.27	31.41	4.71	10.69	-2.43	0.032	9

* Fold represents fold change value which is volume ratio of after the temperature upshift (180 min)/before the temperature upshift. Volume ratio refers to the ratio of the normalized volumes of a pair of spots (the same spot of before and after the temperature upshift), for example, a value of 2.0 represents a two-fold increase while -2.0 represents a two-fold decrease.

^p ^These protein spots are phosphorylated at Ser, Thr and Tyr residues, detected by western blot analysis before the temperature upshift (0 min) and after the temperature upshift (45, 90, 180 min).

Frac/pH range and % cov represent fraction and pH range where proteins were separated in the first dimension, and %coverage, respectively.

Some of the down regulated proteins cannot be clearly visualized on the spot map shown in Additional figures.

ud means the protein expression pattern(s) cannot be clustered.

^t ^Cluster types; (i) in PM fraction, cluster 4 is undetermined-pattern proteins, (ii) in SOL fraction, cluster 18 is sustained tolerance proteins and cluster 8 is undetermined-pattern proteins and (iii) in TM fraction, cluster 12 is resistance proteins, cluster 1, 10, and 17 are adaptation proteins, and cluster 9 and 14 are sustained tolerance proteins.

UvrD/REP helicase plays a critical role in DNA repair by restarting stalled replication forks. It facilitates this process by displacing the RecA protein from DNA [28]. The UvrD/REP helicase is known to be part of the SOS response induced by ultraviolet light (UV), which induces DNA lesions [29]. The down-regulation of this protein suggests that the DNA damage caused by exposure to heat stress can be rescued by a different DNA repair system than the SOS response.

Finally, the level of Δ^9^-desaturase was decreased upon high temperature stress in the photosynthetic membrane of *Spirulina*. This enzyme catalyses the first step of the fatty acid desaturation process in the TM and PM of this cyanobacterium. We observed that the mRNA stability of this gene decreased dramatically in response to high temperature stress [see fig S4; Additional file 2]. Therefore, the reduction in the level of enzyme is likely caused by the decrease in mRNA stability.

### Transcriptional analysis of some differentially expressed proteins

**Figure 1:**
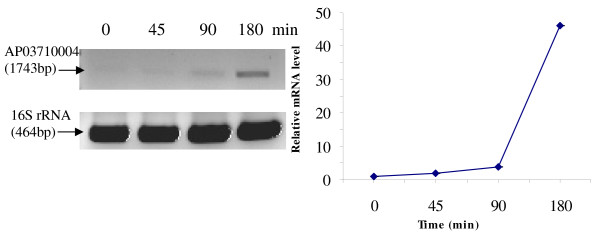
**RT-PCR analysis of the transcriptional level of a differentially expressed protein, AP03710004 - Sensory box/GGDEF family protein (spot no. 599_Sol)**. (Note: Some of the standard deviation values are too small to be seen as error bars.)

**Figure 2:**
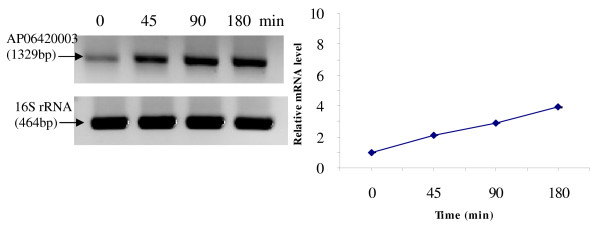
**RT-PCR analysis of the transcriptional level of a differentially expressed protein, AP06420003 - RNA-directed DNA polymerase (spot no. 608_Sol)**. (Note: Some of the standard deviation values are too small to be seen as error bars.)

**Figure 3:**
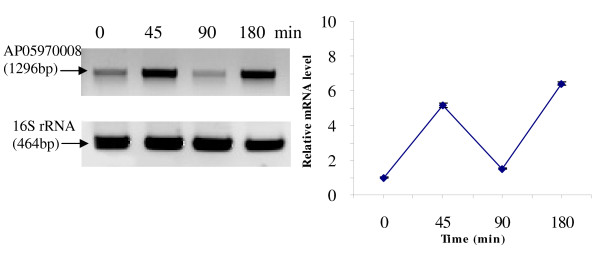
**RT-PCR analysis of the transcriptional level of a differentially expressed protein, AP05970008 - DNA gyrase subunit A (spot no. 1278_Sol)**. (Note: Some of the standard deviation values are too small to be seen as error bars.)

**Figure 4:**
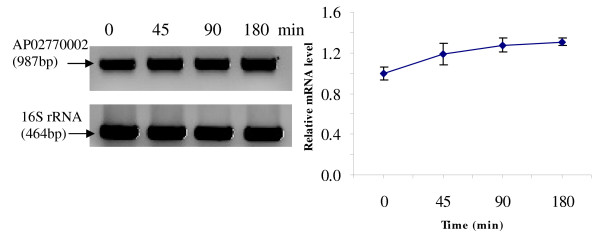
**RT-PCR analysis of the transcriptional level of a differentially expressed protein, AP02770002 - Putative chromosome segregation ATPase (spot no. 1209_Sol)**. (Note: Some of the standard deviation values are too small to be seen as error bars.)

**Figure 5:**
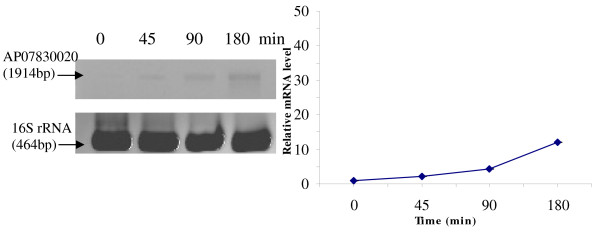
**RT-PCR analysis of the transcriptional level of a differentially expressed protein, AP07830020 - Molecular chaperone DnaK (spot no. 912_TM)**. (Note: Some of the standard deviation values are too small to be seen as error bars.)

**Figure 6:**
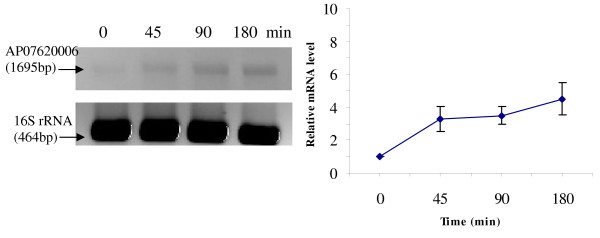
**RT-PCR analysis of the transcriptional level of a differentially expressed protein, AP07620006 - ABC transporter (spot no. 1078_TM)**. (Note: Some of the standard deviation values are too small to be seen as error bars.)

**Figure 7:**
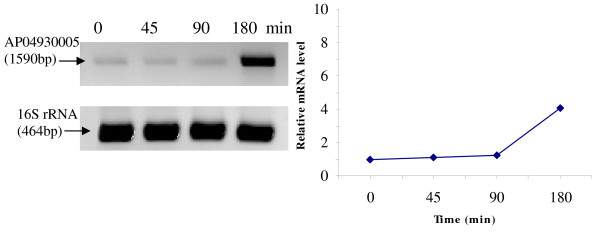
**RT-PCR analysis of the transcriptional level of a differentially expressed protein, AP04930005 - N-6 DNA methylase (spot no. 684_TM)**. (Note: Some of the standard deviation values are too small to be seen as error bars.)

**Figure 8:**
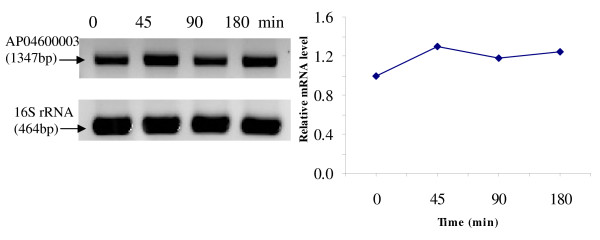
**RT-PCR analysis of the transcriptional level of a differentially expressed protein, AP04600003 - S-adenosyl-L-homocysteine hydrolase (spot no. 2082_TM)**. (Note: Some of the standard deviation values are too small to be seen as error bars.)

**Figure 9:**
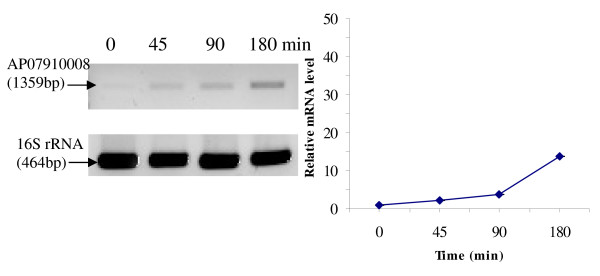
**RT-PCR analysis of the transcriptional level of a differentially expressed protein, AP07910008 - Adenylate cyclase (spot no. 452_TM)**. (Note: Some of the standard deviation values are too small to be seen as error bars.)

**Figure 10:**
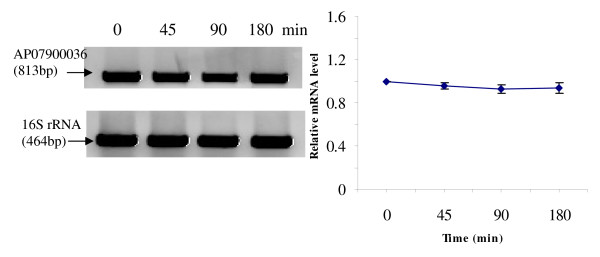
**RT-PCR analysis of the transcriptional level of a differentially expressed protein, AP07900036 - Δ^9 ^desaturase (spot no. 3572_TM)**. (Note: Some of the standard deviation values are too small to be seen as error bars.)

RT-PCR was used to analyze the transcriptional expression levels of some differentially expressed proteins (Fig. 1, 2, 3, 4, 5, 6, 7, 8, 9 and 10) [see fig S5; Additional file 2]. The transcriptional expression patterns of DNA gyrase and chromosome segregation ATPase from the soluble fraction, and ABC transporter, S-adenosyl-L-homocysteine hydrolase and Δ^9 ^desaturase from the thylakoid membrane were well correlated with the protein expression patterns. This correlation suggests that these proteins are likely regulated at the transcriptional level. Interestingly, the Δ^9 ^desaturase gene, the first gene in the fatty acid desaturation process of *Spirulina*, was previously reported to be temperature-independent [9]. However, an earlier study by our group using Northern blot analysis [see fig S4; Additional file 2] demonstrates that this gene is indeed temperature-dependent, in agreement with the results obtained in the present study.

The transcriptional patterns of sensory box/GGDEF and RNA-directed DNA polymerase, both from the soluble fraction, were different than their protein expression patterns. The transcripts of these genes increased throughout the experimental time period (3 hours), while their protein levels initially increased, followed by reduction to steady state protein levels. Importantly, phosphorylation was detected on both of these proteins. This suggests that the post-translational modification might play a role in the function of these proteins in response to the high temperature stress.

In the thylakoid membrane fraction, the transcription patterns of DnaK and N-6 DNA methylase (adenine specific) were not well correlated with their protein expression patterns, and phosphorylation was not detected for these proteins. These results suggest that these proteins might be regulated at either the post-transcriptional level or the post-translational level (except phosphorylation), although further investigation will be required to confirm this hypothesis. There are some interesting facts related to these three proteins in the photosynthetic membrane of *Spirulina*. It has been reported that the N-6 DNA methylase uses S-adenosyl-methionine as a methyl-donor for its DNA-methylation reaction in plants [30]. It should be noted that the S-adenosyl-L-homocysteine hydrolase, the enzyme responsible for regeneration of S-adenosyl-methionine [31,32], was also up-regulated in the same fraction.

In *Synechococcus *sp. PCC7942, photosystem II (PSII) is drastically deactivated at 40°C [33]. The chaperone DnaK (Hsp70) is present in plant-chloroplast as a component of multi-chaperone complex [34] and plays a critical role in photoprotection and repair of PSII during and after photoinhibition [35,36]. Thus, it is expected that this chaperone was up-regulated in response to the high temperature stress in *Spirulina*, although its regulation should be further investigated.

Adenylate cyclase, which is known to localize in the thylakoid membrane of cyanobacteria, plays a key role in cAMP biosynthesis [37]. The level of cAMP is regulated by red/far red light and thus adenylate cyclase works in association with phytochrome [37], an up-regulated protein found in *Spirulina*-TM. These proteins are part of the cAMP-dependent light signaling cascade. Adenylate cyclase has also been reported to be regulated at the post-translational level by ligand binding, protein binding and phosphorylation [37]. Together, our results demonstrate the association between high temperature response and the light signaling cascade.

### Clustering of protein expression patterns

**Figure 11:**
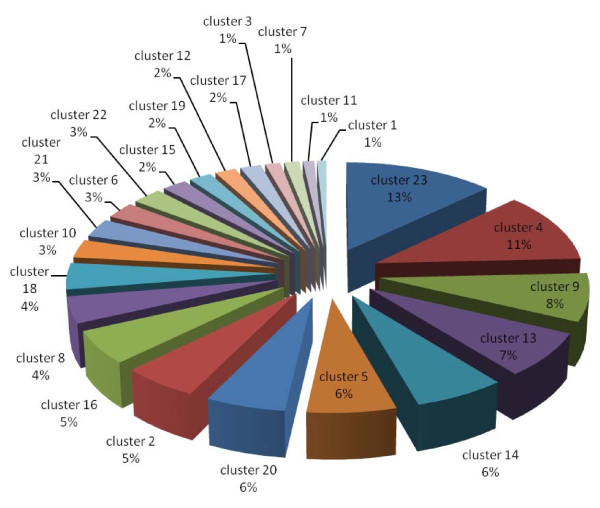
**Pie charts representing percentage of each protein cluster classified by the expression pattern of all significant differentially expressed proteins in the plasma membrane fraction *(clusters 7, 11, 12 and 21 are resistance proteins, cluster 22 is adaptation protein, clusters 1, 5, 6, 14, 15, 16, 18, 20 and 23 are sustained proteins, and clusters 2, 3, 4, 8, 9, 10, 13, 17 and 19 are undetermined protein trends)***.

**Figure 12:**
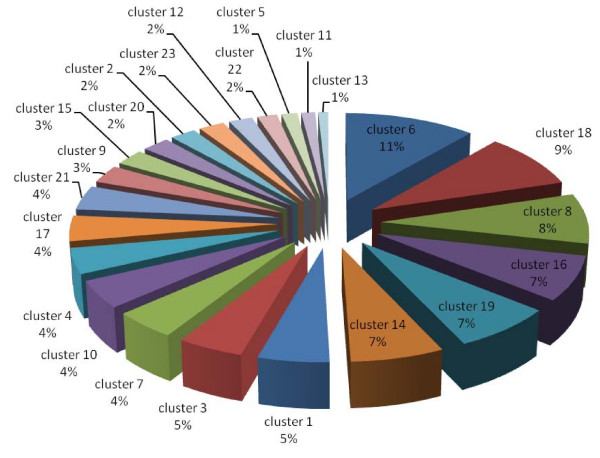
**Pie charts representing percentage of each protein cluster classified by the expression pattern of all significant differentially expressed proteins in the soluble fraction *(clusters 9, 17 and 20 are resistance proteins, clusters 7, 12 and 21 are adaptation proteins, clusters 1, 2, 3, 6, 16, 18 and 19 are sustained proteins, and clusters 4, 5, 8, 10, 11, 13, 14, 15, 22 and 23 are undetermined protein trends)***.

**Figure 13:**
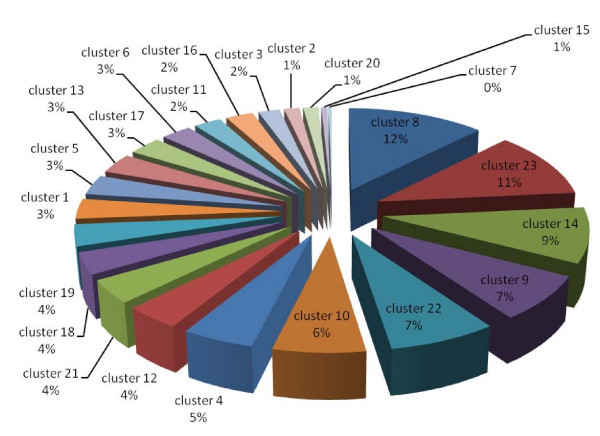
**Pie charts representing percentage of each protein cluster classified by the expression pattern of all significant differentially expressed proteins in the thylakoid membrane fraction *(clusters 4, 11, 12, 16, 19 and 20 are resistance proteins, clusters 1, 10 and 17 are adaptation proteins, clusters 2, 6, 8, 9, 13, 14, 18, 21, 22 and 23 are sustained proteins, and clusters 3, 5, 7 and 15 are undetermined protein trends)***.

The proteins with significantly differential expression in each subcellular fraction were clustered, based on their expression patterns [see fig S6; Additional file 2]. According to Lacerda et al., the expression patterns in response to stress can be classified into three major groups: resistance, adaptation and sustained tolerance [38]. The results shown in Fig. 11, 12 and 13 demonstrate that the majority of proteins in every subcellular fraction belong to the sustained tolerance expression pattern. If all differentially expressed proteins are set as 100%, the percentages of the resistance, adaptation and sustained tolerance groups are: (*i*) 7%, 3% and 46% in the PM fraction, (*ii*) 9%, 10%, 46% in the soluble fraction and (*iii*) 18%, 12% and 58% in the TM fraction, respectively. It should be noted that some patterns do not fit into any categories, and these patterns were mostly found in the PM fraction. Moreover, the plasma membrane, where the environmental changes are first encountered, is the only site where the resistance proteins are present at a significantly higher level than the adaptation proteins.

Site-specific DNA methyltransferase (cytosine-specific) is the only resistance protein that was identified in this study. The level of this protein initially increased and subsequently decreased in the TM fraction (Table 4). DNA methylase is involved in the DNA repair system, and it shows the same expression pattern in response to cadmium stress, which is known to induce DNA-damage [38]. Most of the two component signal transduction systems, stress-related proteins and proteins involved in DNA-damage and DNA-repair are classified in the sustained tolerance group (Fig. 11). This suggests the critical role of these proteins in the tolerance to high temperature stress in *Spirulina*. It is noteworthy that the resistance proteins (short-term only response) were present at a significantly higher level (2-fold) in the thylakoid membrane than in the other two fractions (Fig. 13). Additionally, adaptation proteins (long-term only response) were found at a higher level in the soluble and the thylakoid membrane fractions (Fig. 12 and 13) than the plasma membrane fraction (Fig. 11).

### Potential protein-protein interactions

**Figure 14:**
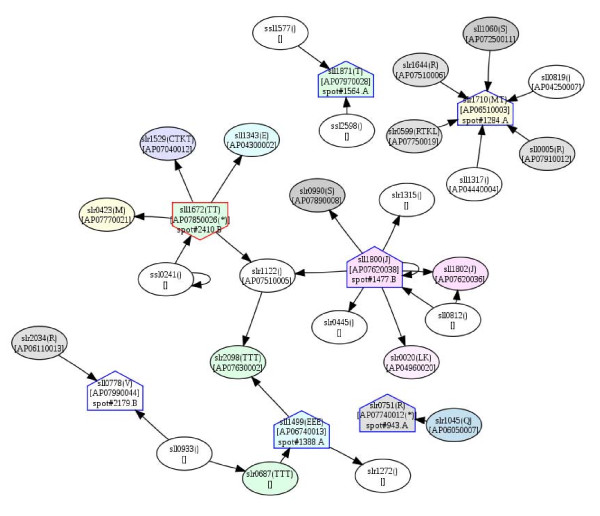
**Predicted protein-protein interaction network based on differentially expressed proteins identified in this work, constructed by using the available data from Cyanobase and the *Spirulina *genome database**. The networks show protein-protein interaction partners in the plasma membrane fraction. The symbols, ⌂ and its reversion, represent the up- and down-regulated proteins identified in this study, respectively. The letters A and B after spot numbers in the nodes represent the pH ranges of 3-10 and 4-7 in the first dimension of the 2D-DIGE, respectively.

**Figure 15:**
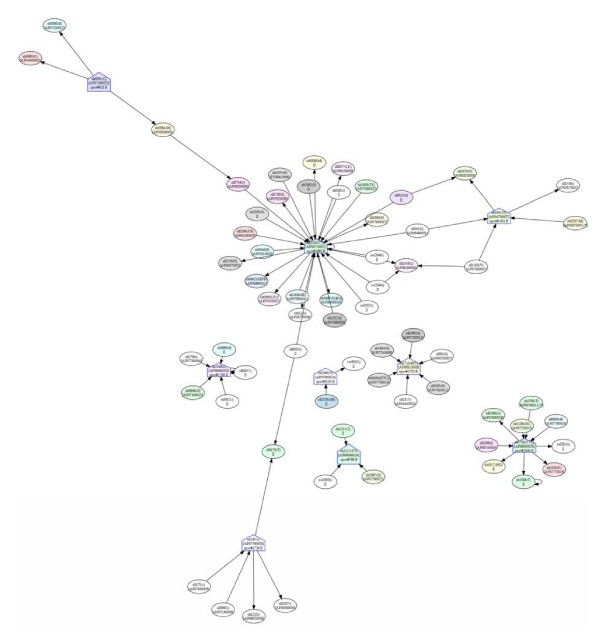
**Predicted protein-protein interaction network based on differentially expressed proteins identified in this work, constructed by using the available data from Cyanobase and the *Spirulina *genome database**. The networks show protein-protein interaction partners in the soluble fraction. The symbols, ⌂ and its reversion, represent the up- and down-regulated proteins identified in this study, respectively. The letters A and B after spot numbers in the nodes represent the pH ranges of 3-10 and 4-7 in the first dimension of the 2D-DIGE, respectively.

**Figure 16:**
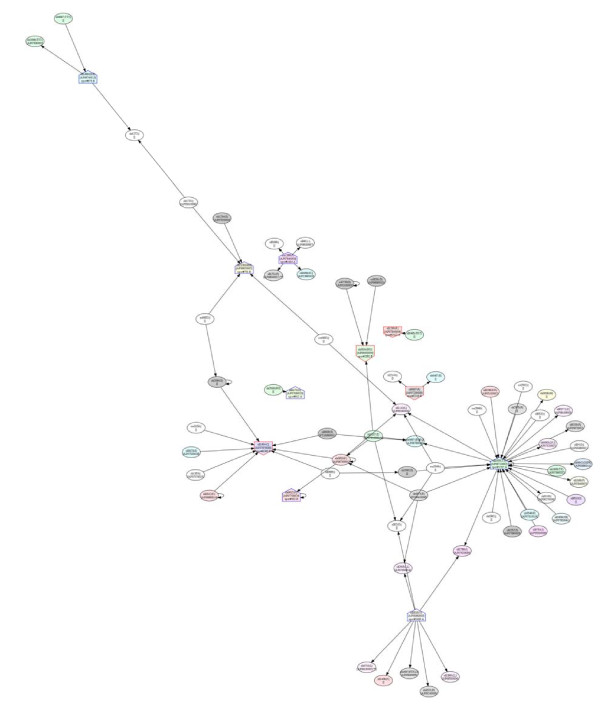
**Predicted protein-protein interaction network based on differentially expressed proteins identified in this work, constructed by using the available data from Cyanobase and the *Spirulina *genome database**. The networks show protein-protein interaction partners in the thylakoid membrane fraction. The symbols, ⌂ and its reversion, represent the up- and down-regulated proteins identified in this study, respectively. The letters A and B after spot numbers in the nodes represent the pH ranges of 3-10 and 4-7 in the first dimension of the 2D-DIGE, respectively.

Several differentially expressed proteins identified in this study can be mapped onto the PPI network available on Cyanobase (Fig. 14, 15 and 16). The potential PPIs shown in the three subcellular fractions represent interesting linkages or cross-talks among the three cellular compartments. For example, in the PM fraction, two component system sensory histidine kinase (spot#1564), ABC transporter (spot#2179), ferredoxin-glutamate synthase (spot#1388) and carboxypeptidase (spot#1284) show interactions with the photosynthetic system. In the soluble fraction, the phosphorylated form of multi-sensor signal transduction histidine kinase (spot#1883) interacts with several periplasmic proteins. However, in the TM fraction, the same protein was found in the non-phosphorylated form. The interactions found in the thylakoid membrane also show communication with the other two fractions.

Additionally, PPI networks clearly demonstrate the linkage between high temperature stress and nitrogen and ammonia assimilation in *Spirulina*. It is well established that photosynthesis and nitrate reduction are closely related in cyanobacteria and plants, via the nitrate reductase requirement of photoreduced ferredoxin [39,40]. In response to heat stress, inhibition of photosynthesis and nitrate reductase was observed. Moreover, it was reported by Rajaram and Apte [40] that a Hsp60 family protein, Cpn60, which is induced by heat stress and stabilized by nitrogen supplementation, either from nitrate or ammonia, is essential for the thermal stability of these vital metabolic processes.

## Conclusion

The differentially expressed proteins identified in the subcellular fractions of *Spirulina *in response to high temperature stress can be functionally classified into 5 major groups: two component systems, stress-related proteins, DNA damage/DNA repair system, translational machinery and proteins with conserved motifs. The transcriptional expression levels of several proteins were studied by RT-PCR. Several of the differentially expressed proteins, such as DNA gyrase and ABC transporter, were regulated at the transcriptional level. Some proteins, such as sensory box/GGDEF domain and RNA-directed DNA polymerase, were found to be regulated at the post-translational level. Finally, other proteins, such as DnaK and adenylate cyclase, were found to be regulated at the post-transcriptional level.

All the differentially expressed proteins were subjected to protein clustering, based on their expression pattern in the three cellular compartments. The clustering data assists in grouping the up- or down-regulated proteins into three major trends: resistance proteins, adaptation proteins and sustained tolerance proteins. The majority of the differentially expressed proteins from all subcellular fractions were found to be sustained tolerance proteins, suggesting the critical role of these proteins in the tolerance of *Spirulina *to high temperature stress. A group of resistance proteins (short-term only expression) in the photosynthetic membrane was present at 2-fold higher levels than in either of the other two fractions. This is well correlated with the report [33] that photosynthetic systems are rapidly affected by high temperature (40°C) in the present of light.

According to the data obtained from the PPI network construction, the cross-talk and linkages between the three cellular compartments, via protein-protein interactions, were substantial. The data give clear evidence that the nitrogen and ammonia assimilation processes are affected by exposure to heat stress.

In terms of applications, the present proteomic analysis and PPI network construction are part of an attempt to control and manipulate conditions to maximize polyunsaturated fatty acid (PUFA) biosynthesis in this cyanobacterium. Taken together with the data obtained in our cold-shock response study of *S. platensis *[11,13], several proteins involved in fatty acid biosynthesis, such as histidine kinases, (3R)-hydroxymyristoyl- [acyl-carrier-protein]-dehydratase or FabZ, acyl carrier protein (ACP) and Δ^9^-desaturase, were revealed to be differentially expressed. The knowledge obtained can be applied at the industrial level, for manipulation of PUFA production, as well as in future studies of various aspects of *Spirulina*. In addition to valuable product biosynthesis in *Spirulina*, the results from the PPI network are beneficial to the functional annotation of some *Spirulina platensis*-ORFs. For example, AP07850026 (spot#2410), annotated as a two component system, could possibly be functionally annotated to a two component system in the Nar family, due to its interaction with a protein involved with nitrogen assimilation. Finally, future proteomic analysis of *Spirulina *will involve analyzing the complete proteome of *Spirulina *by combining the techniques of 2D-PAGE and liquid chromatography-tandem mass spectrometry (LC-MS/MS).

## Competing interests

The authors declare that they have no competing interests.

## Authors' contributions

AH carried out the proteome analysis and the protein-protein interaction analysis, conceived of the study, and participated in its design and coordination. MS participated in the proteome analysis. RY participated in the proteome analysis. JS carried out the potential protein-protein interaction construction and others statistical analysis. PK carried out the molecular genetic studies. SC participated in the design of the study. MT participated in the design of the study. All authors read and approved the final manuscript.

## Supplementary Material

Additional file 1 **Details on primers conditions used in RT-PCR experiments**. The table provides details on primers conditions used in RT-PCR experiments. Click here for file

Additional file 2 **Additional figures 1-6**. The data provided represent spot map of 2D-DIGE of all protein fractions, quantitative analysis of protein, of which mRNAs were analyzed by RT-PCR, and protein clustering based on their expression level. Click here for file
