# Supplementary material for: Subcellular proteomic characterization of the high-temperature stress response of the cyanobacterium Spirulina platensis
Source: Proteome Sci. 2009 Sep 2;7:33. doi: 10.1186/1477-5956-7-33 (PMC2743650; doi:10.1186/1477-5956-7-33)
Supplement: Additional file 1 — Details on primers conditions used in RT-PCR experiments. The table provides details on primers conditions used in RT-PCR experiments. [file 1477-5956-7-33-S1.doc]

**Table S1** Details on primers conditions used in RT-PCR experiments

| Sample name | Primer name | Oligonucleotide primers  5′ 3′ | RT-PCR condition |  |
| --- | --- | --- | --- | --- |
| AP03710004 | AP03710004F  AP03710004R | ATGAAAAAAATTCTGATTATTGAAGATGAAAG  TCAATAATTGGGTAGGTTAAAATCAGGATC | 45°C 45min. | 1 cycle (Reverse transcription) |
|  |  |  | 95°C 2 min.  95°C 45 sec.  50°C 45 sec  72°C 2 min | 35 cycles  (PCR amplification) |
|  |  |  | 72°C 10min | 1 cycle (final extension) |
| AP06420003 | AP06420003F  AP06420003R | ATGACGAAAGCGAGTTTAAAGAAAGGG  CTAAACGTTTCGCACTATGAGGTCTACTT | 45°C 45min. | 1 cycle (Reverse transcription) |
|  |  |  | 95°C 2 min.  95°C 45 sec.  55°C 45 sec  72°C 2 min | 35 cycles  (PCR amplification) |
|  |  |  | 72°C 10min | 1 cycle (final extension) |
| AP05970008 | AP05970008F  AP05970008R | ATGTTAGCCCTAGTTAACGGAAAACC  TCAGTTATTGACTTCTTGAAGTTCAACGTC | 45°C 45min. | 1 cycle (Reverse transcription) |
|  |  |  | 95°C 2 min.  95°C 45 sec.  52°C 45 sec  72°C 2 min | 35 cycles  (PCR amplification) |
|  |  |  | 72°C 10min | 1 cycle (final extension) |
| AP02770002 | AP02770002F  AP02770002R | ATGAGCAAATTAGCATCTTTAGAAACCCAG  TTATTGATTGTTACGATTCCCAGAAAACG | 45°C 45min. | 1 cycle (Reverse transcription) |
|  |  |  | 95°C 2 min.  95°C 45 sec.  50°C 45 sec  72°C 1 min | 35 cycles  (PCR amplification) |
|  |  |  | 72°C 10min | 1 cycle (final extension) |
| AP07830020 | AP07830020F  AP07830020R | ATGGGAAAAGTAGTTGGAATTGACTTAGGA  TTATTTAGATTCGGTGAAGTCCGCATC | 45°C 45min. | 1 cycle (Reverse transcription) |
|  |  |  | 95°C 2 min.  95°C 45 sec.  50°C 45 sec  72°C 2 min | 35 cycles  (PCR amplification) |
|  |  |  | 72°C 10min | 1 cycle (final extension) |
| AP07620006 | AP07620006F  AP07620006R | ATGCTGCGACTAGAACATA  CTATCTACCCTTAGCCGCTG | 45°C 45min. | 1 cycle (Reverse transcription) |
|  |  |  | 95°C 2 min.  95°C 45 sec.  55°C 45 sec  72°C 2 min | 35 cycles  (PCR amplification) |
|  |  |  | 72°C 10min | 1 cycle (final extension) |
| AP04930005 | AP04930005F  AP04930005R | ATGCCAAAGTCCAAGAAAACCCAAT  TCATAGCCCTAACCTCCTCAAATTTTC | 45°C 45min. | 1 cycle  (Reverse transcription) |
|  |  |  | 95°C 2 min.  95°C 45 sec.  52°C 45 sec  72°C 2 min | 35 cycles  (PCR amplification) |
|  |  |  | 72°C 10min | 1 cycle (final extension) |
| AP04600003 | AP04600003F  AP04600003R | TTGGGTGCCCCCATAATTTG  TTAGTCAGTGCCAACAGTCCAGG | 45°C 45min. | 1 cycle (Reverse transcription) |
|  |  |  | 95°C 2 min.  95°C 45 sec.  52°C 45 sec  72°C 2 min | 35 cycles  (PCR amplification) |
|  |  |  | 72°C 10min | 1 cycle (final extension) |
| AP07910008 | AP07910008F  AP07910008R | ATGAAATGGATTAAGTTGAATAATGGC  TCAATATAACACTTCAAAGACTTCTATCT | 45°C 45min. | 1 cycle (Reverse transcription) |
|  |  |  | 95°C 2 min.  95°C 45 sec.  50°C 45 sec  72°C 2 min | 35 cycles  (PCR amplification) |
|  |  |  | 72°C 10min | 1 cycle (final extension) |
| AP07900036 | AP07900036F  AP07900036R | ATGACGATTGCAACTGAAACCAAACT  CTAGTTTTCCACCAATTTAACTTTTTTTAGC | 45°C 45min. | 1 cycle (Reverse transcription) |
|  |  |  | 95°C 2 min.  95°C 45 sec.  50°C 45 sec  72°C 1 min | 35 cycles  (PCR amplification) |
|  |  |  | 72°C 10min | 1 cycle (final extension) |
