# Supplementary material for: Subcellular proteomic characterization of the high-temperature stress response of the cyanobacterium Spirulina platensis
Source: Proteome Sci. 2009 Sep 2;7:33. doi: 10.1186/1477-5956-7-33 (PMC2743650; doi:10.1186/1477-5956-7-33)
Supplement: Additional file 2 — Additional figures 1-6. The data provided represent spot map of 2D-DIGE of all protein fractions, quantitative analysis of protein, of which mRNAs were analyzed by RT-PCR, and protein clustering based on their expression level. [file 1477-5956-7-33-S2.doc]

**Additional Figure Legends**

**Figure S1** Fluorescently labeled two dimensional gel maps of *S. platensis* C1 cellular fractions using IPG-strips with a pH range of (a) 3-10 and (b) 4-7 in the first dimension of plasma membrane fraction. The spots labeled with protein spot ID are the differentially expressed proteins after data filtering as described in *Materials and Methods*.

**Figure S2** Fluorescently labeled two dimensional gel maps of *S. platensis* C1 cellular fractions using IPG-strips with a pH range of (a) 3-10 and (b) 4-7 in the first dimension of soluble fraction. The spots labeled with protein spot ID are the differentially expressed proteins after data filtering as described in *Materials and Methods*.

**Figure S3** Fluorescently labeled two dimensional gel maps of *S. platensis* C1 cellular fractions using IPG-strips with a pH range of (a) 3-10 and (b) 4-7 in the first dimension of thylakoid membrane fraction. The spots labeled with protein spot ID are the differentially expressed proteins after data filtering as described in *Materials and Methods*.

**Figure S4** The stability study of the three desaturase mRNAs under various growth temperatures in dark conditions was carried out in the presence of 150 µg ml^-1^ rifampicin. Five micrograms of total RNA were loaded into each lane. Northern blot analysis showing the mRNA levels of *desC* and the graph showed relative changes in the mRNA levels of *desC*. (♦ and ■ in the graphs represent the level of the designated mRNA at 40^o^C and 35^o^C, respectively.)

**Figure S5** Quantitative evaluation of protein expression levels of the 10 proteins, encoded by the genes subjected to data validation by RT-PCR, before (0 min) and after (45, 90, 180 min) the temperature upshift.

(A spot represents abundance of a protein after normalization from an experiment. A line connects mean values of each experimental group (0, 45, 90 and 180 min).)

**Figure S6** Protein clustering based on the expression pattern of all significant differentially expressed proteins in the three subcellular fractions, (a) plasma membrane fraction, (b) soluble fraction and (c) thylakoid membrane fraction.

Figure S1a

pI 3 9.3


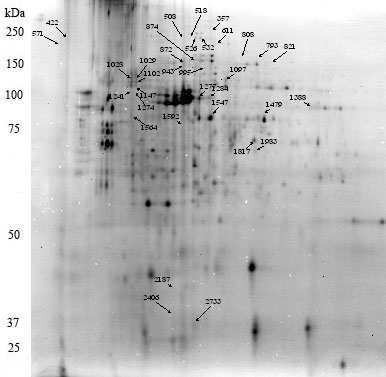


Figure S1b

pI 4.3 6.5


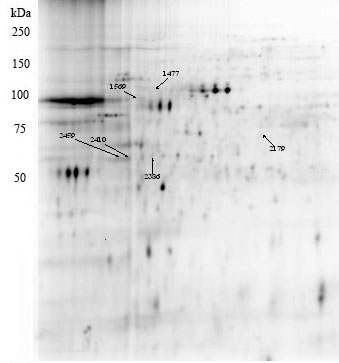


Figure S2a

pI 3.2 9.3


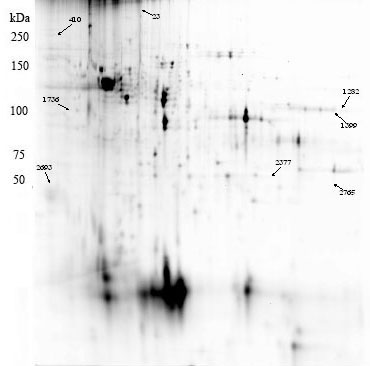


Figure S2b

pI 4 6.6


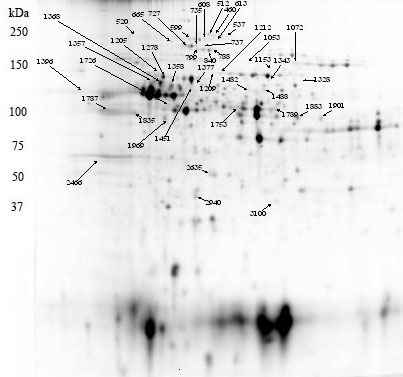


Figure S3a

pI 3 8.7


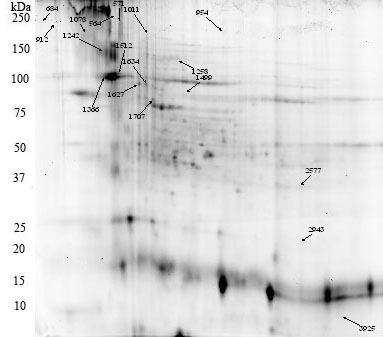


Figure S3b

pI 4.3 7


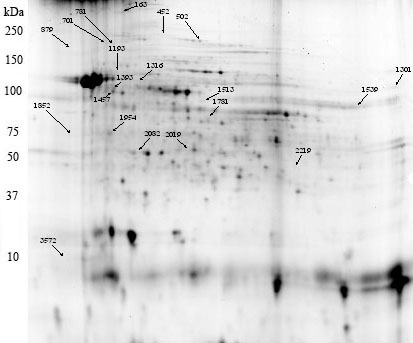


**Figure S4**

**0 5 10 15 20 30 60 90 120 min**

**40^o^C**

**35^o^C**


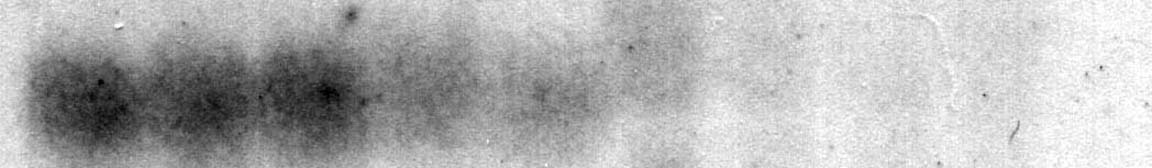

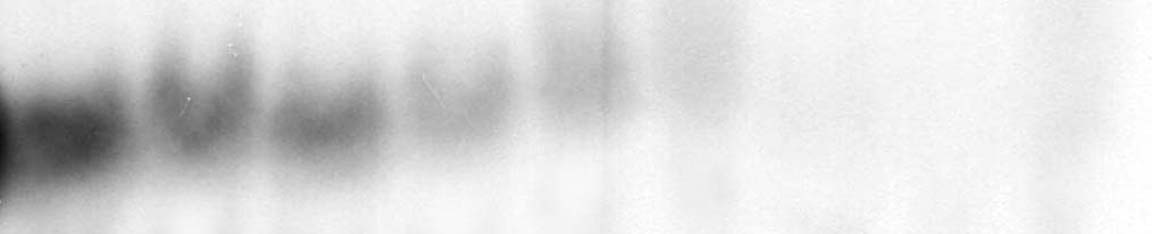


**mRNA level (relative %)**

**Time (min)**

**Figure S5**

AP03710004_ spot#599_Sol pH4-7

AP06420003_ spot#608_Sol pH4-7

AP05970008_ spot#1278_Sol pH4-7

AP02770002_ spot#1209_Sol pH4-7

AP07830020_ spot#912_TM pH3-10

AP07620006_ spot#1078_TM pH3-10

AP04930005_ spot#684_TM pH3-10

AP04600003_ spot#2082_TM pH4-7

AP07910008_ spot#452_TM pH4-7

AP07900036_ spot#3572_TM pH4-7

**Figure S6a**

**
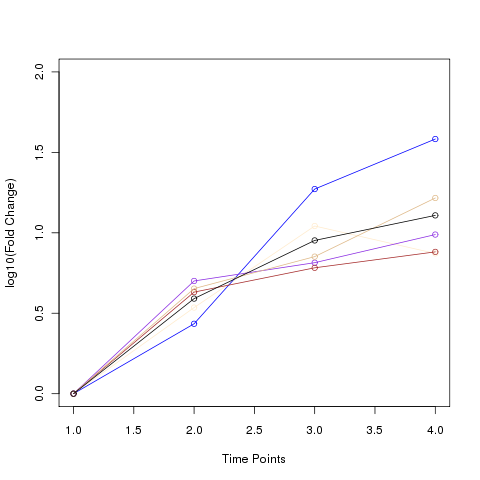

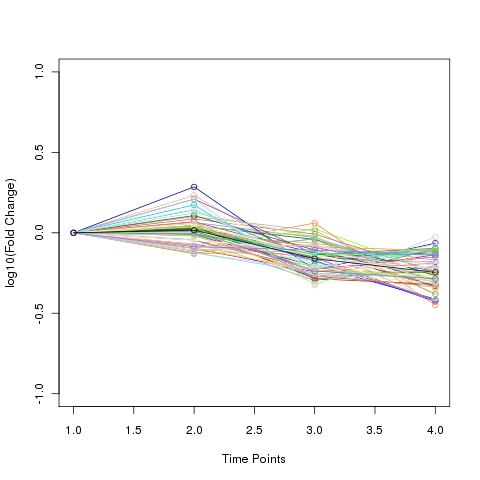

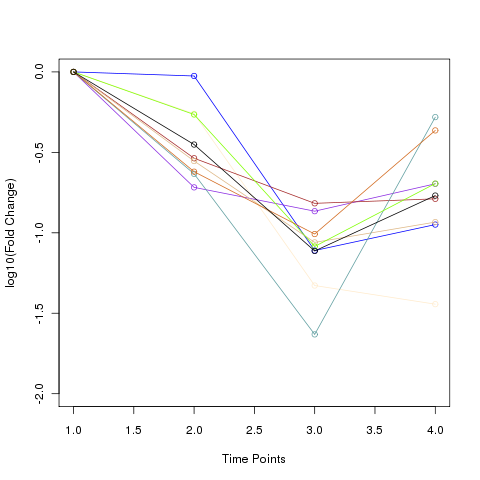

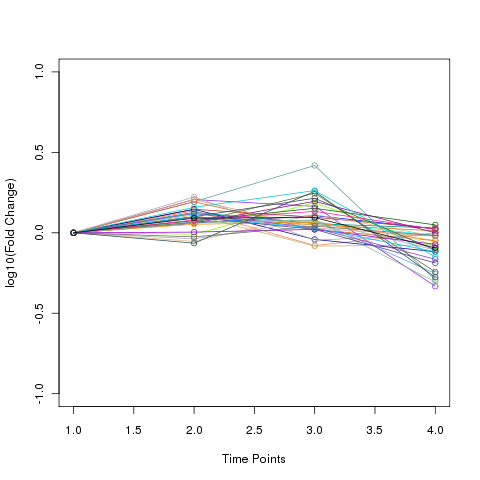

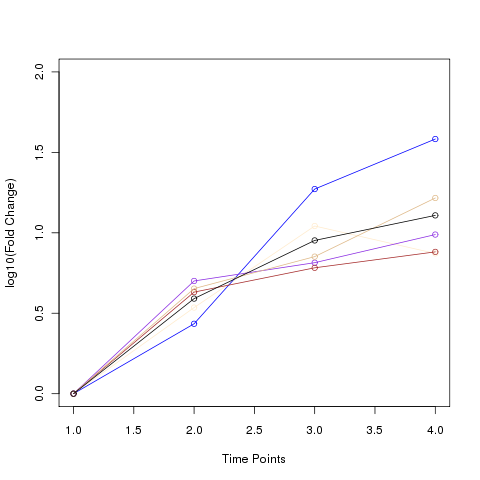

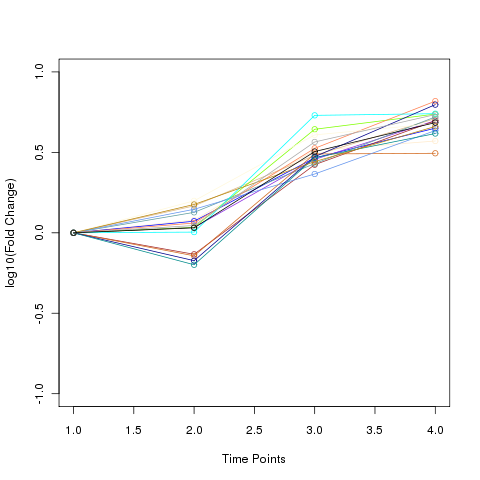
**

Cluster 1

Cluster 2

Cluster 3

Cluster 4

Cluster 5

Cluster 6

**
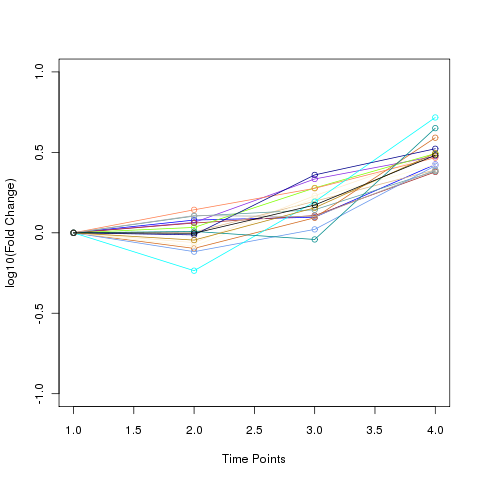

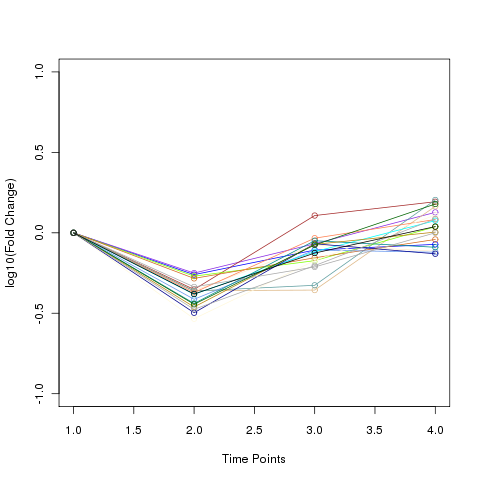

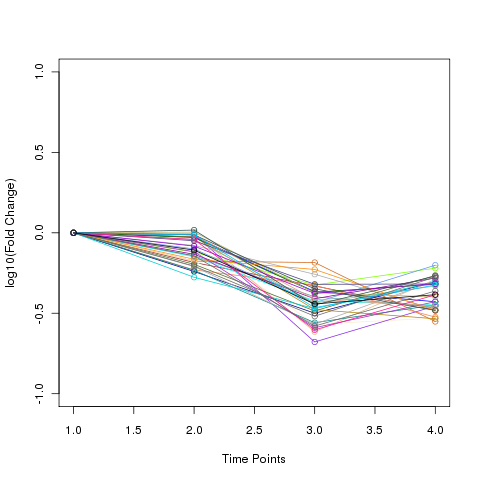

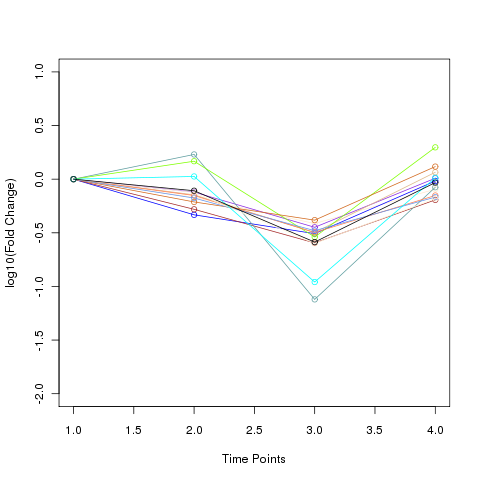

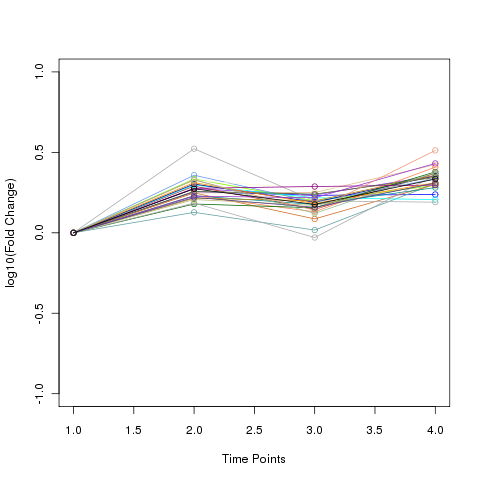

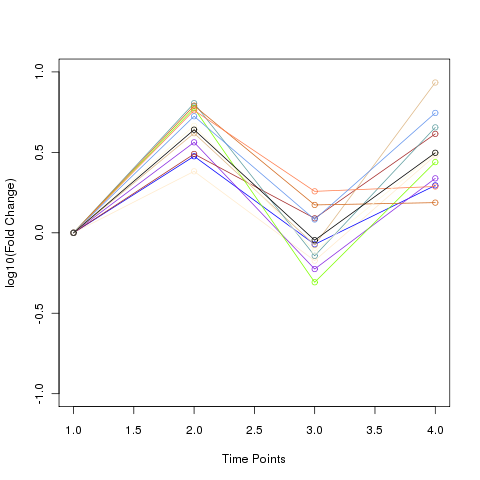

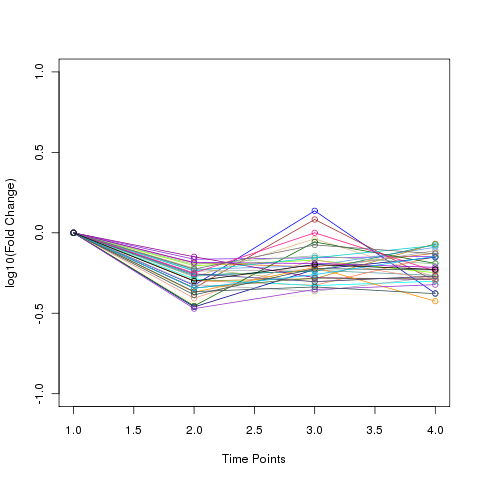

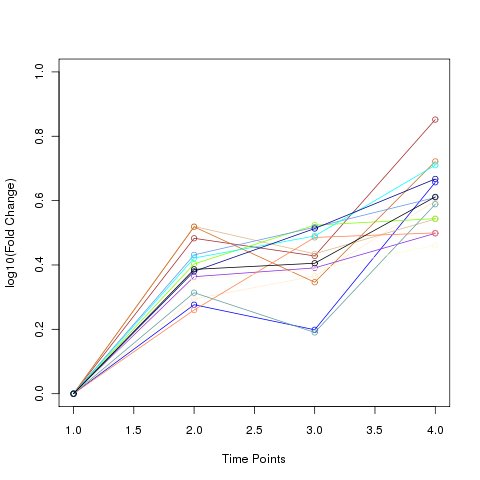

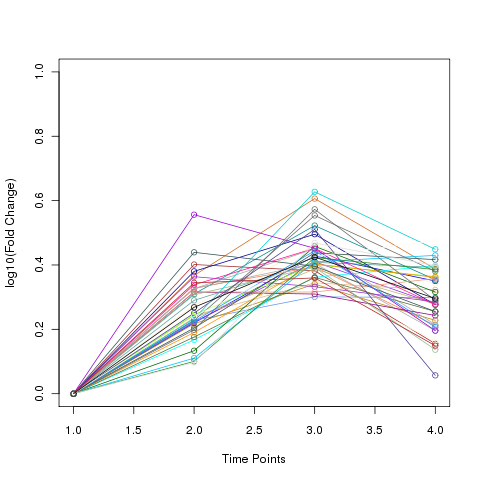

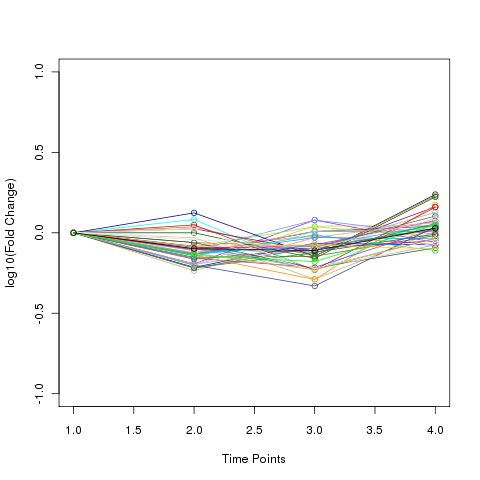

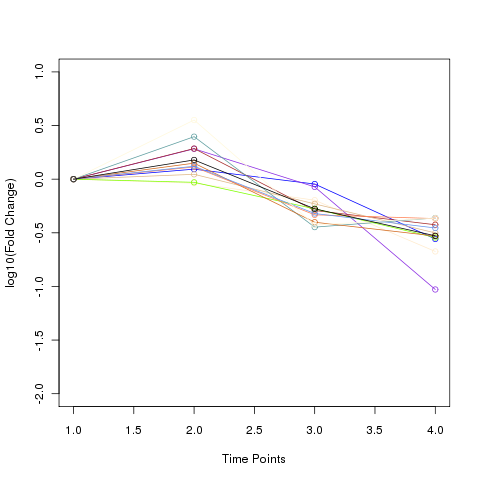

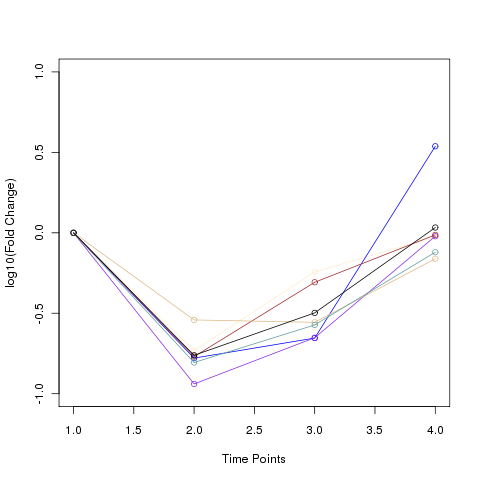

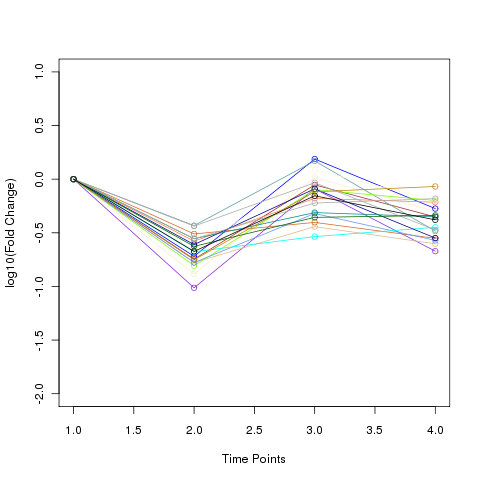

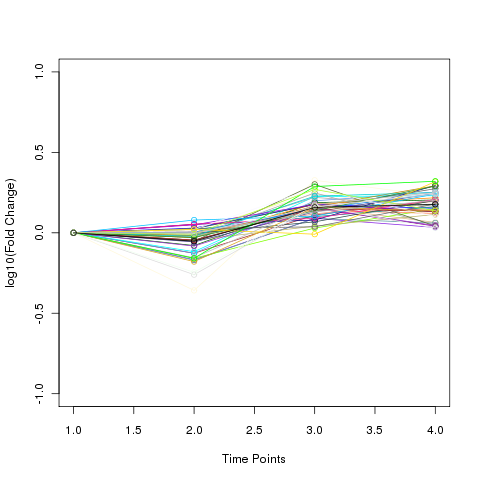

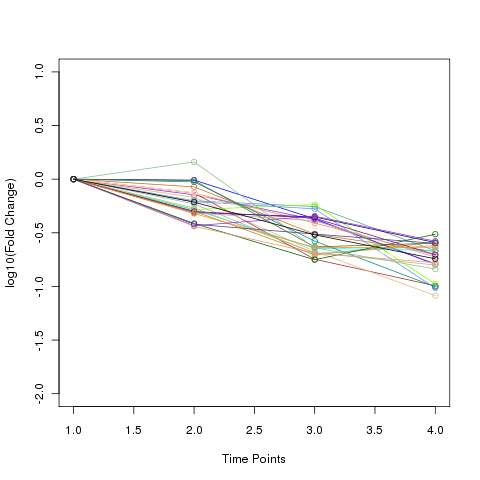

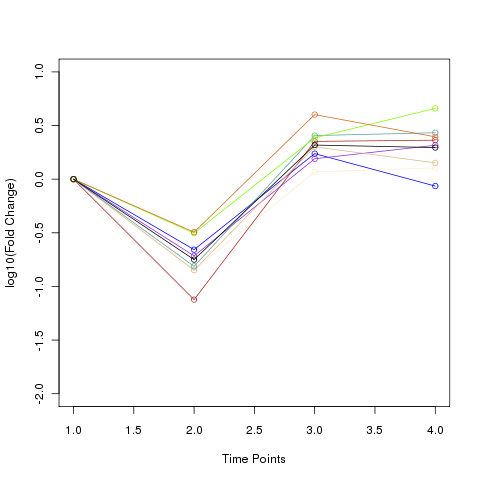

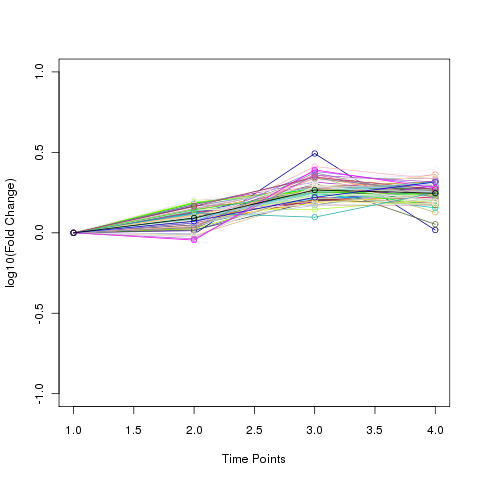
**

Cluster 7

Cluster 8

Cluster 9

Cluster 10

Cluster 11

Cluster 12

Cluster 13

Cluster 14

Cluster 15

Cluster 16

Cluster 17

Cluster 18

Cluster 19

Cluster 20

Cluster 21

Cluster 22

Cluster 23

**Figure S6b**

**
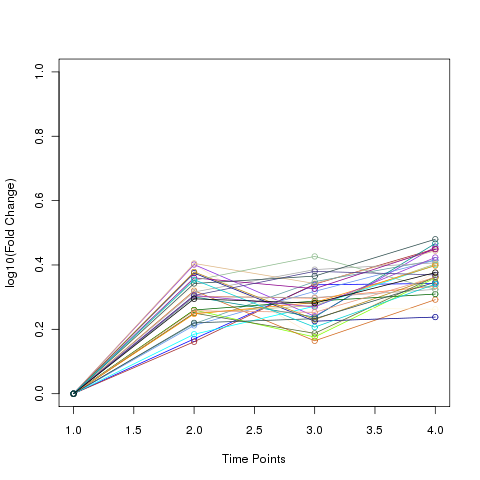
**

Cluster 1

**
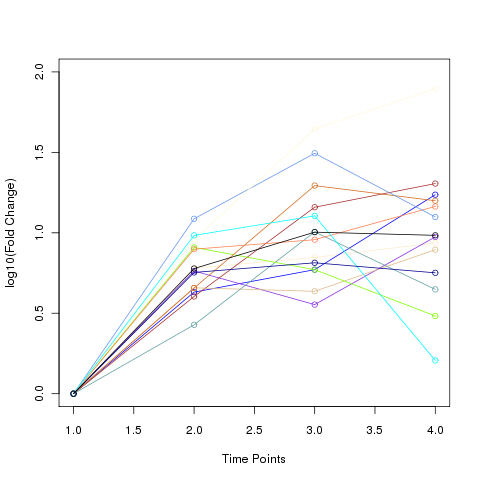
**

Cluster 2

**
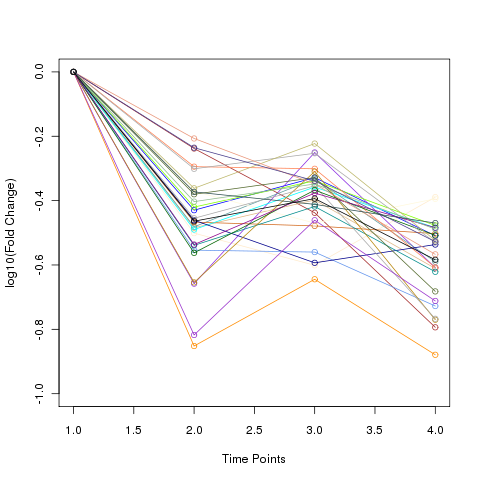
**

Cluster 3

**
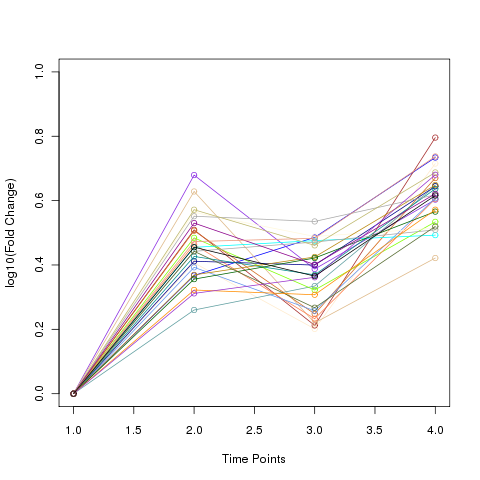
**

Cluster 4

**
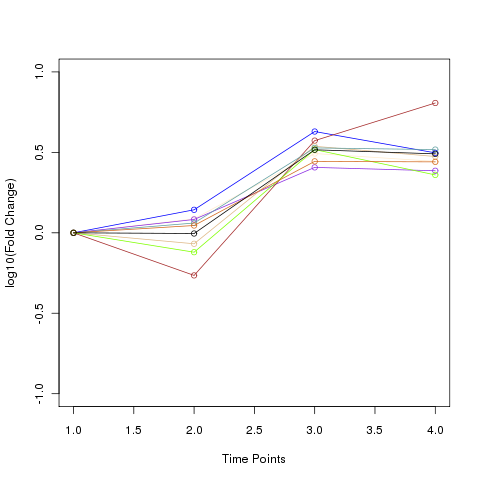
**

Cluster 5

**
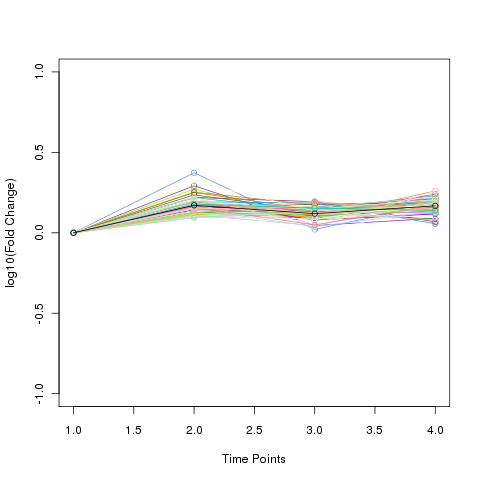
**

Cluster 6

**
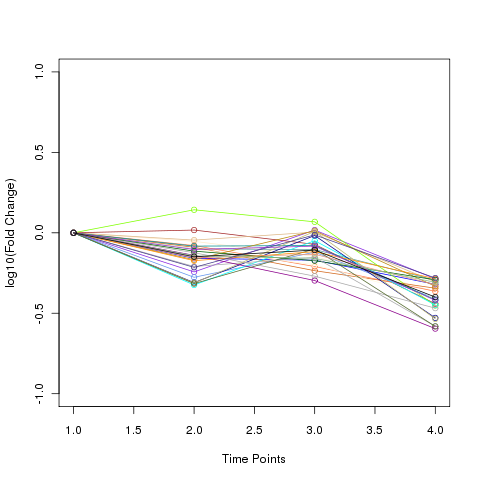
**

Cluster 7

**
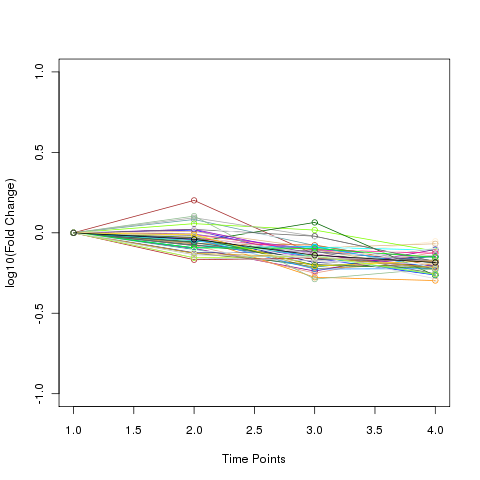
**

Cluster 8

**
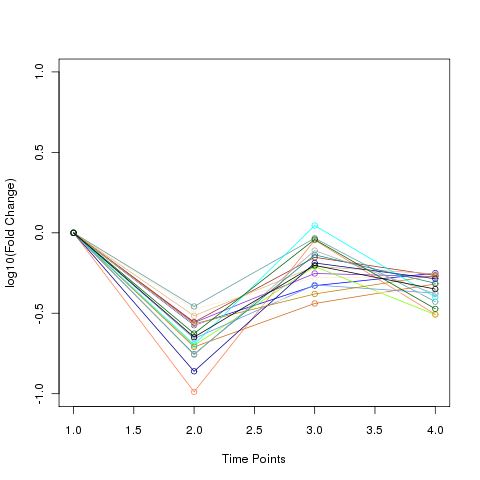
**

Cluster 9

**
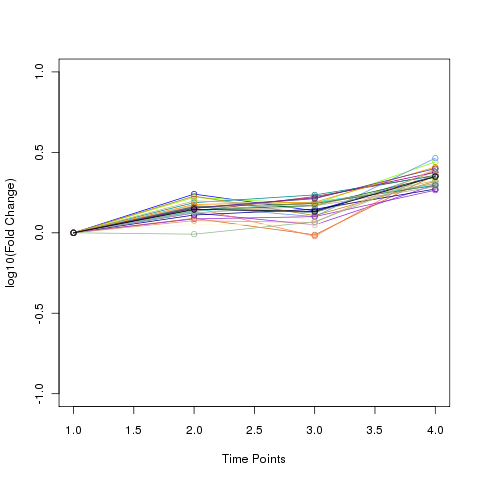
**

Cluster 10

**
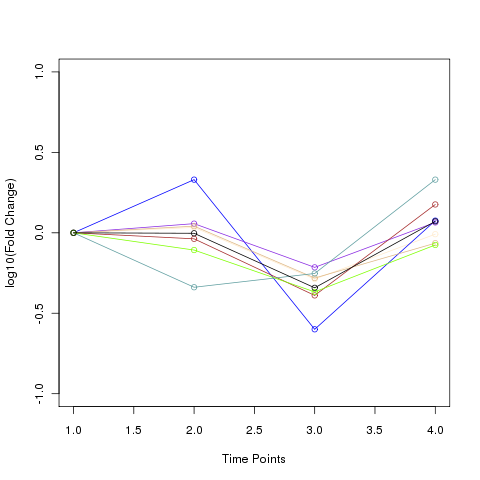
**

Cluster 11

**
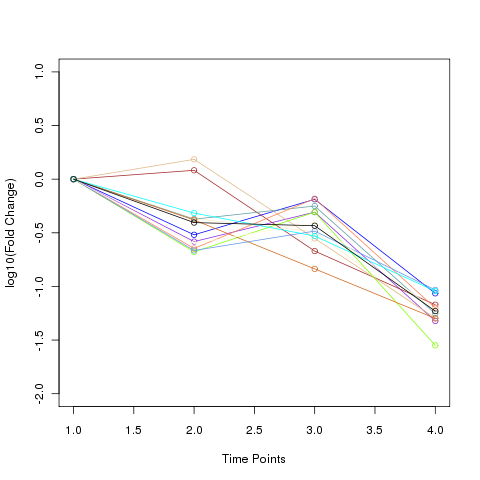
**

Cluster 12

**
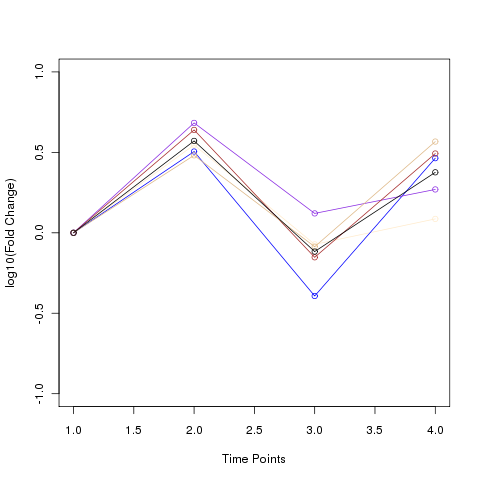
**

Cluster 13

**
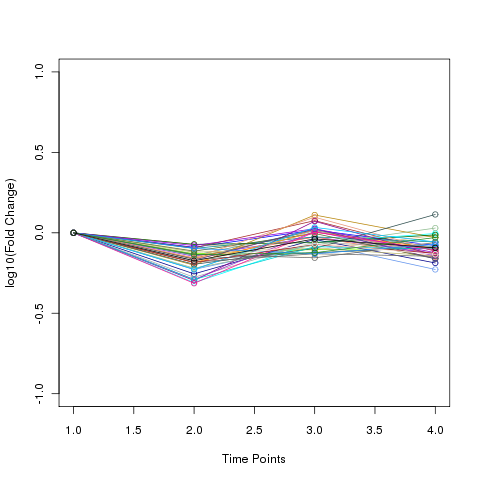
**

Cluster 14

**
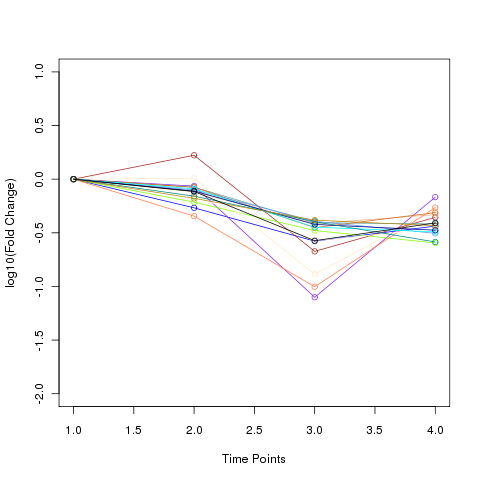
**

Cluster 15

**
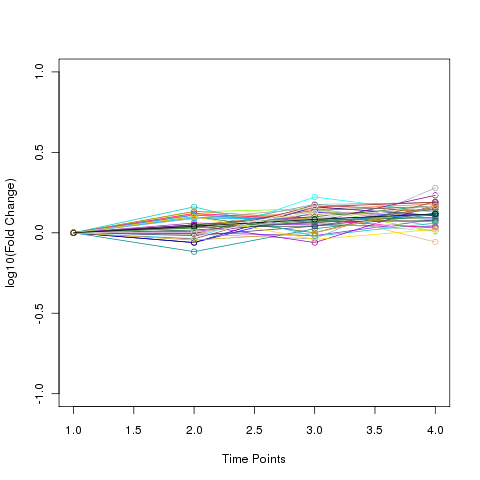
**

Cluster 16

**
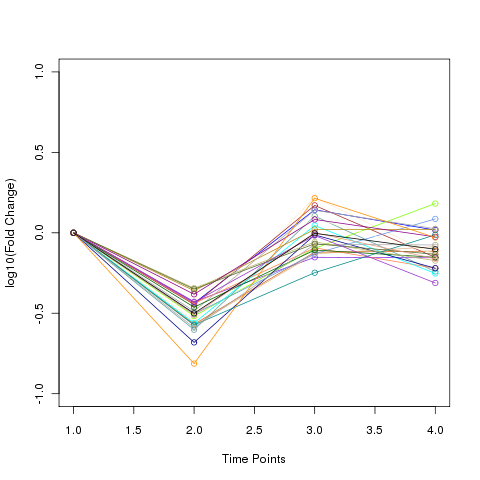
**

Cluster 17

**
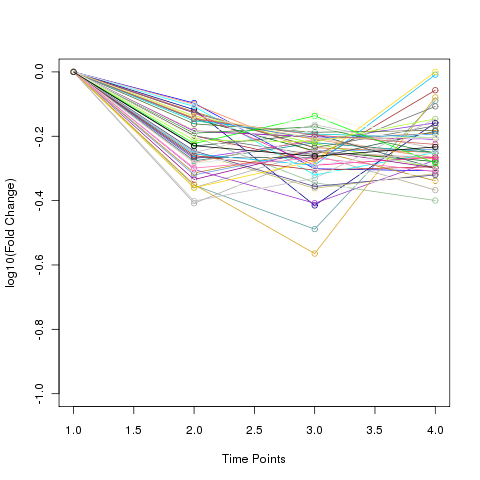
**

Cluster 18

**
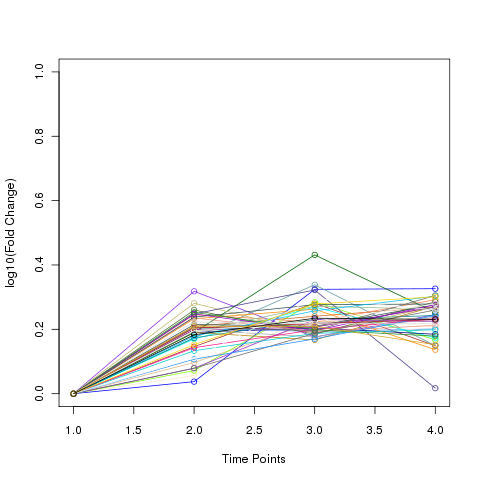
**

Cluster 19

**
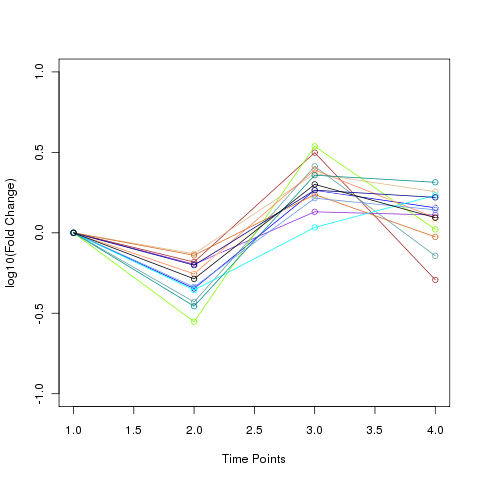
**

Cluster 20

**
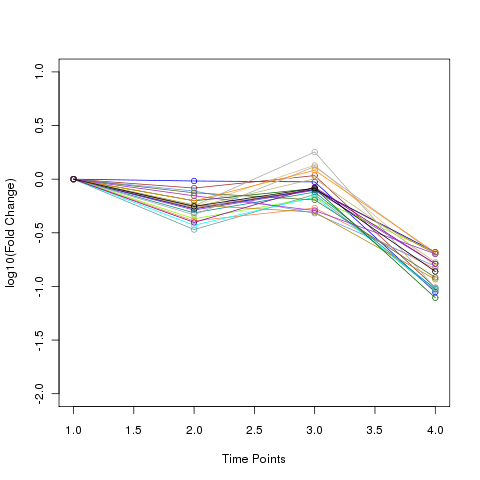
**

Cluster 21

**
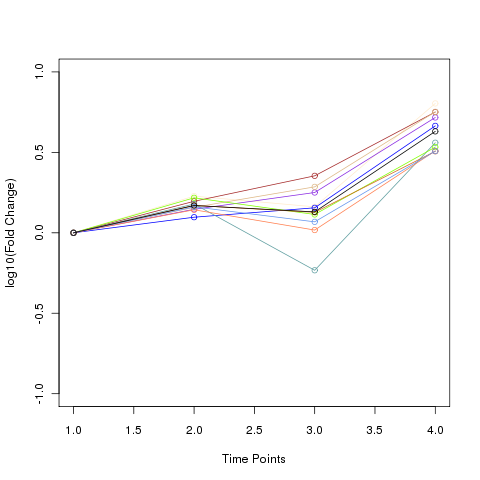
**

Cluster 22

**
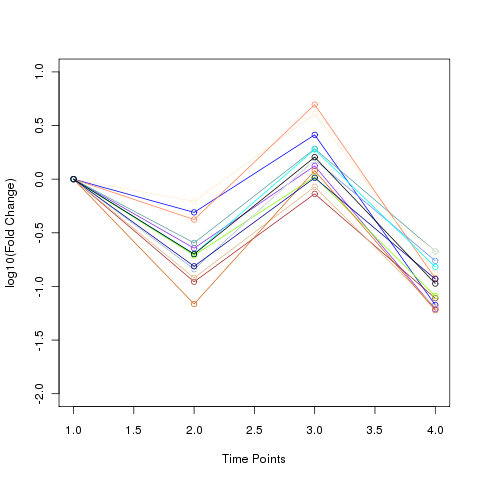
**

Cluster 23

**Figure S6c**

**
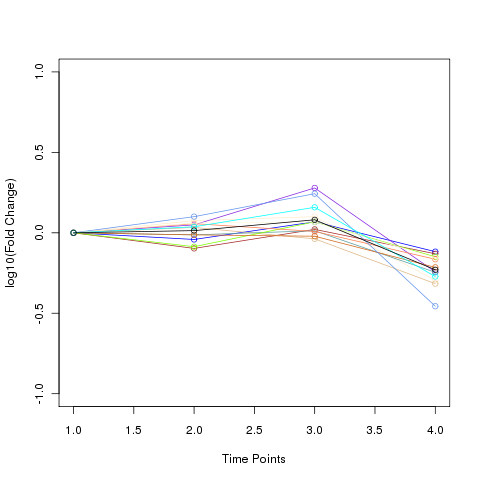
**

Cluster 1

**
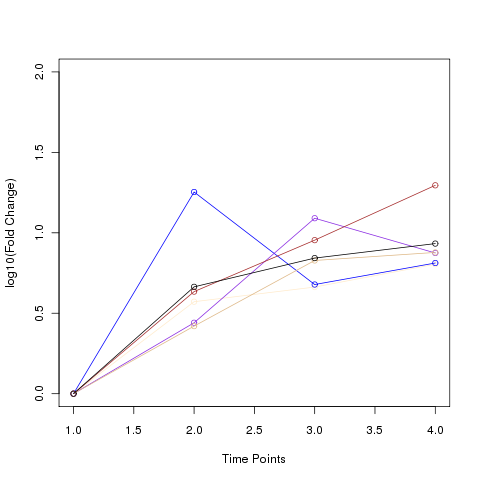
**

Cluster 2

**
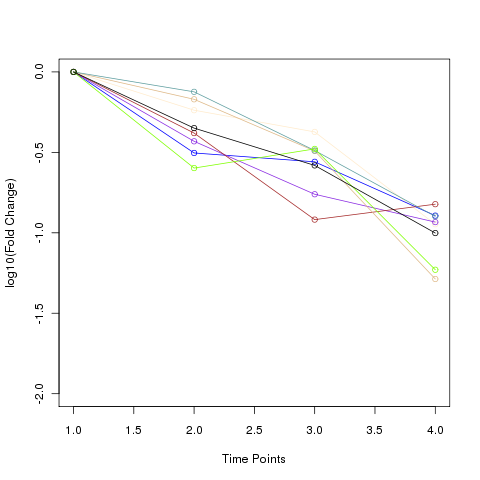
**

Cluster 3

**
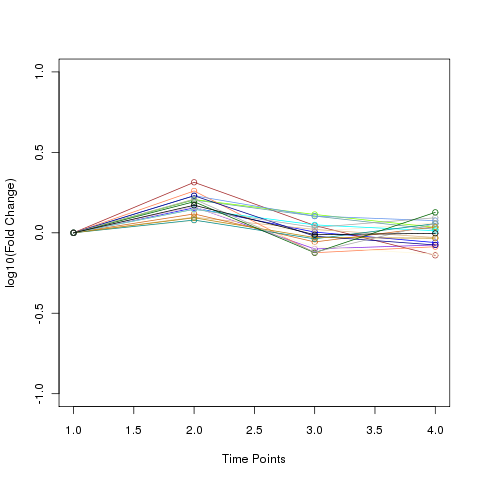
**

Cluster 4

**
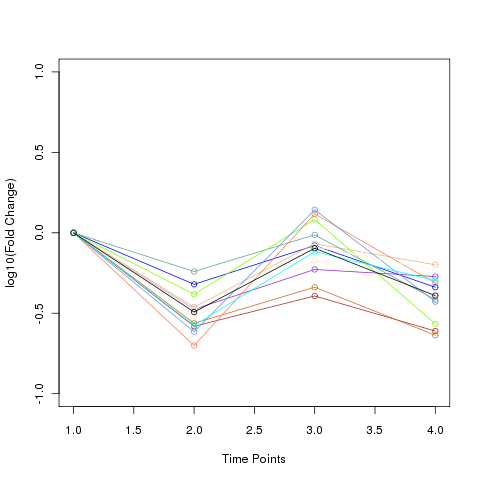
**

Cluster 5

**
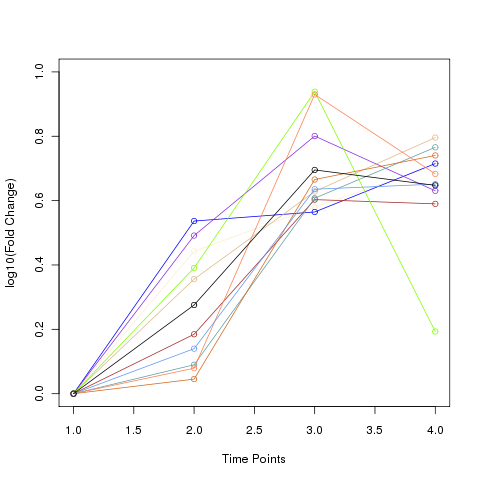
**

Cluster 6

**
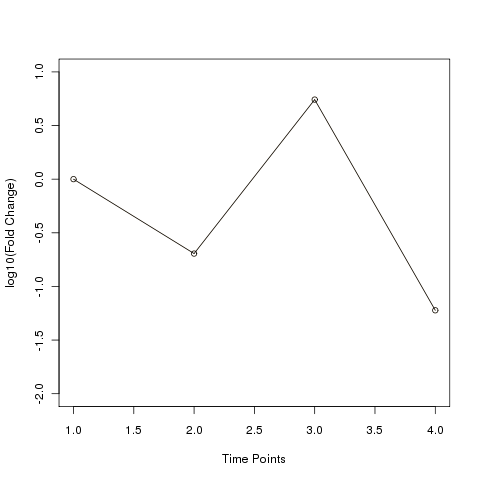
**

Cluster 7

**
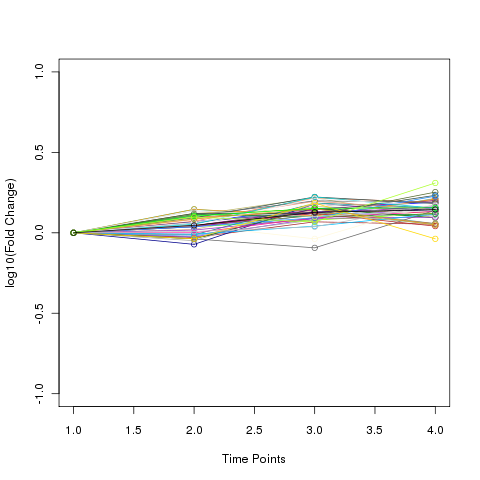
**

Cluster 8

**
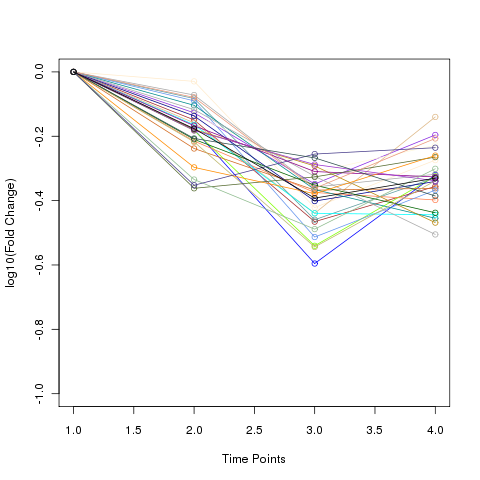
**

Cluster 9

**
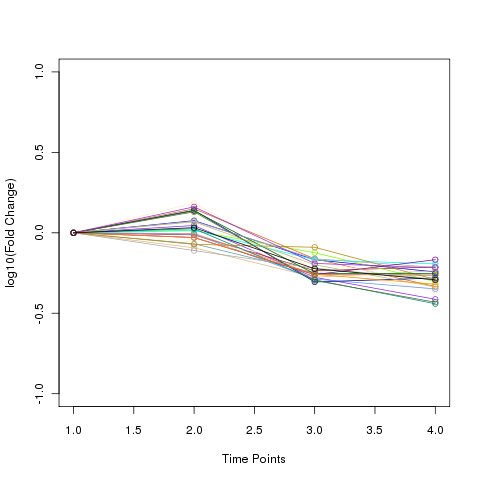
**

Cluster 10

**
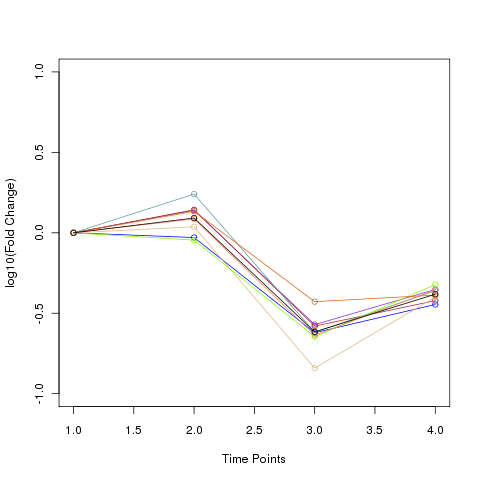
**

Cluster 11

**
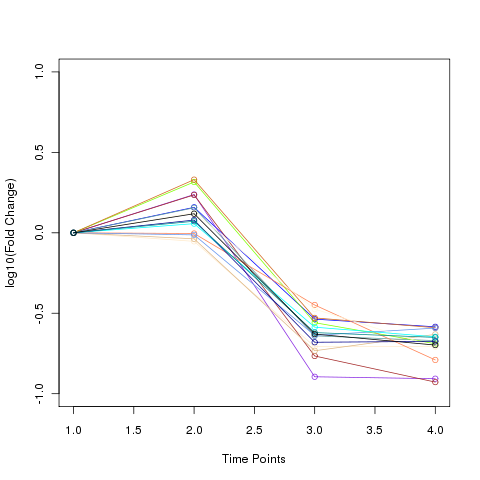
**

Cluster 12

**
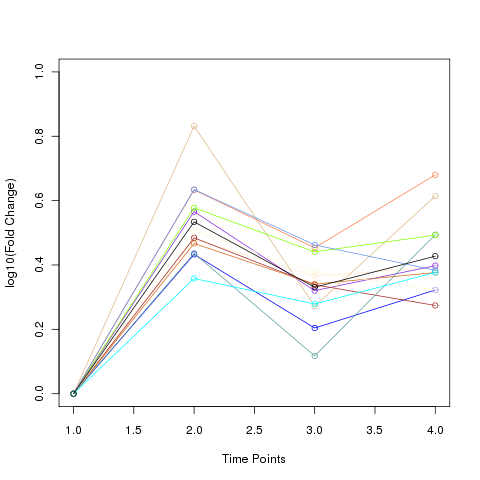
**

Cluster 13

**
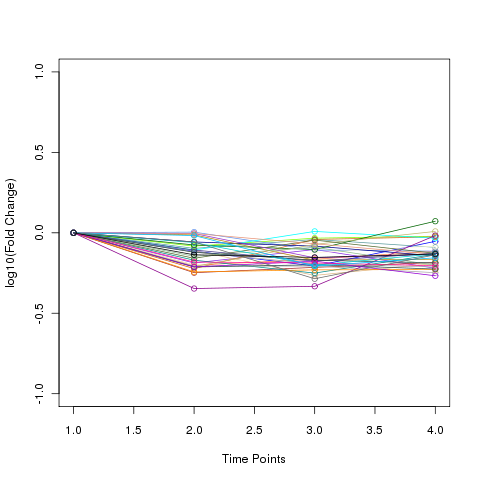
**

Cluster 14

**
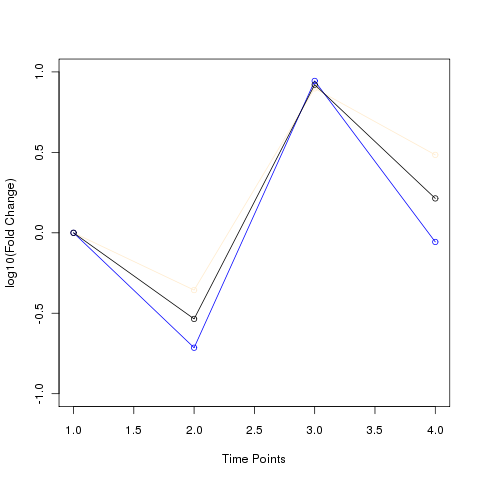
**

Cluster 15

**
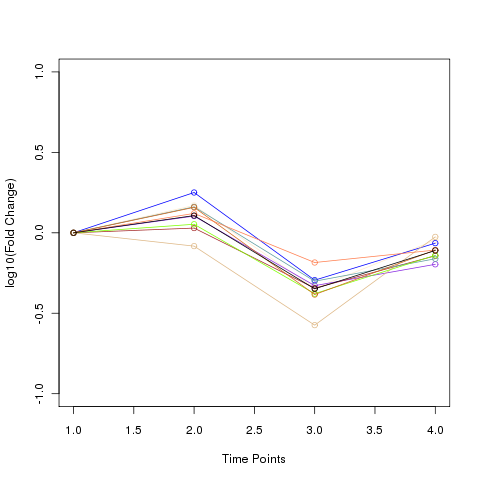
**

Cluster 16

**
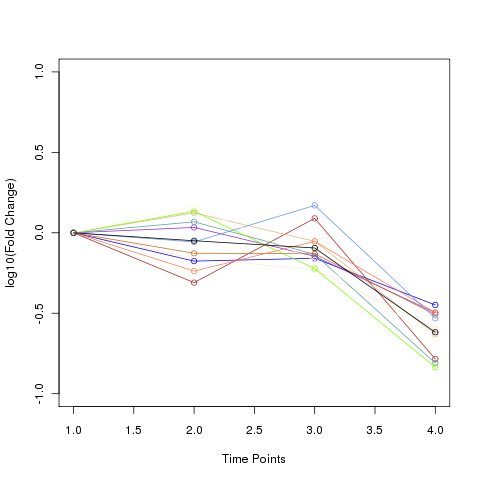
**

Cluster 17

**
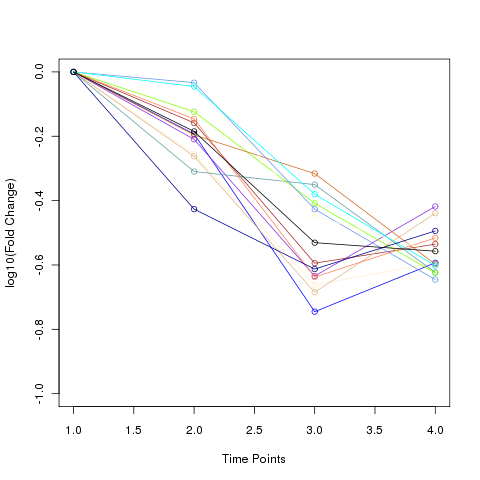
**

Cluster 18

**
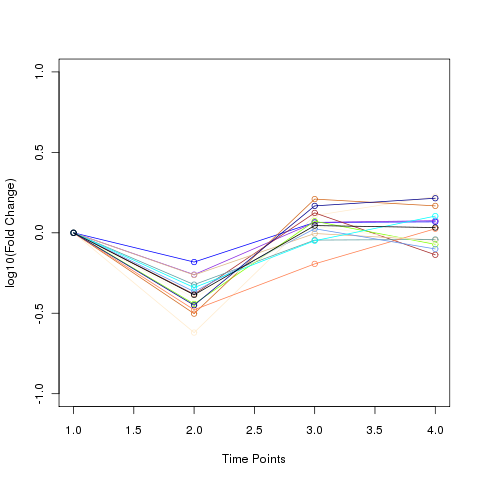
**

Cluster 19

**
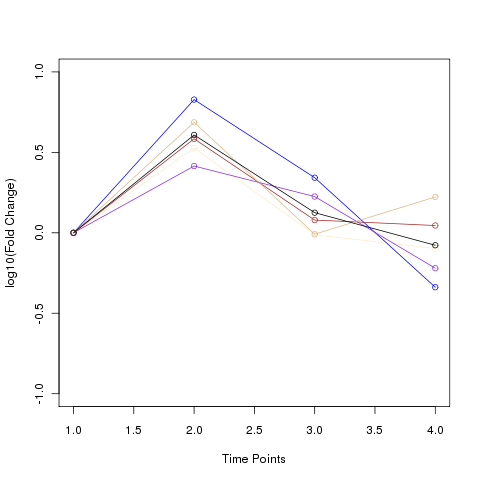
**

Cluster 20

**
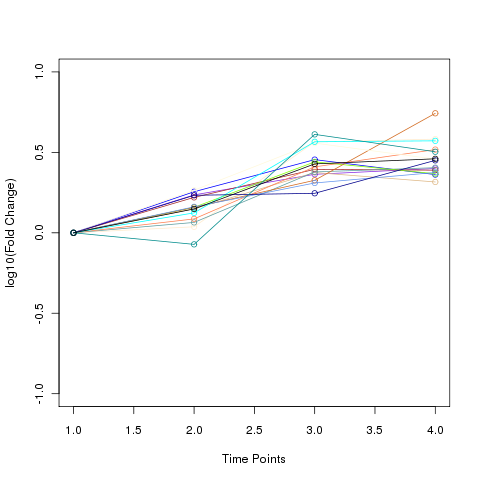
**

Cluster 21

**
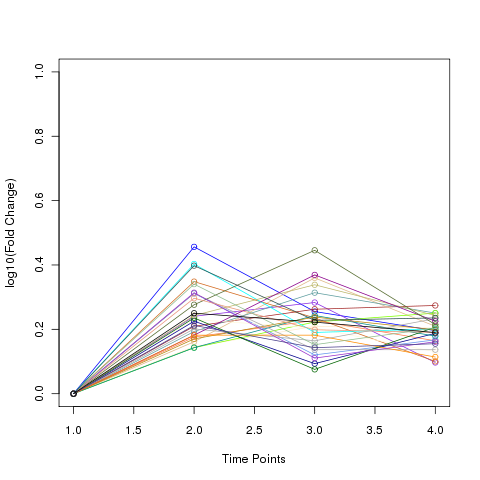
**

Cluster 22

**
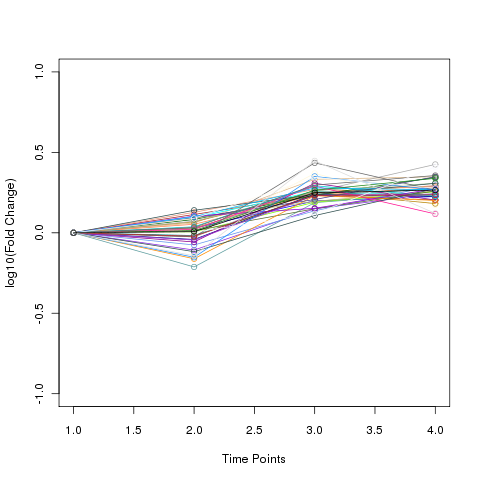
**

Cluster 23
